# Rashba effect on finite temperature magnetotransport in a dissipative quantum dot transistor with electronic and polaronic interactions

**DOI:** 10.1038/s41598-023-32750-x

**Published:** 2023-04-04

**Authors:** Kuntal Bhattacharyya, Debika Debnath, Ashok Chatterjee

**Affiliations:** 1 School of Physics, University of Hyderabad, Hyderabad, 500046 India; 2 Present Address: Department of Physics, GITAM University, Hyderabad, India

**Keywords:** Nanoscience and technology, Physics

## Abstract

The Rashba spin–orbit coupling induced quantum transport through a quantum dot embedded in a two-arm quantum loop of a quantum dot transistor is studied at finite temperature in the presence of electron–phonon and Hubbard interactions, an external magnetic field and quantum dissipation. The Anderson-Holstein-Caldeira-Leggett-Rashba model is used to describe the system and several unitary transformations are employed to decouple some of the interactions and the transport properties are calculated using the Keldysh technique. It is shown that the Rashba coupling alone separates the spin-up and spin-down currents causing zero-field spin-polarization. The gap between the up and down-spin currents and conductances can be changed by tuning the Rashba strength. In the absence of a field, the spin-up and spin-down currents show an opposite behaviour with respect to spin–orbit interaction phase. The spin-polarization increases with increasing electron–phonon interaction at zero magnetic field. In the presence of a magnetic field, the tunneling conductance and spin-polarization change differently with the polaronic interaction, spin–orbit interaction and dissipation in different temperature regimes. This study predicts that for a given Rashba strength and magnetic field, the maximum spin-polarization in a quantum dot based device occurs at zero temperature.

## Introduction

Spintronics has emerged in the last few decades as a very fascinating area of modern condensed matter physics due to its potential use in manipulating electron spin^1,2^ to control spin current. The spin–orbit (SO) interaction which is one of the key elements of low-dimensional spintronics physics has been studied by many research groups^3–14^. These studies have been motivated by the pioneering work of Datta and Das on spin field-effect-transistor^14^. Molecular transistor is another branch which has received so much attention thanks to Aviram et al.^15^ who fabricated the first model of Single Molecular Transistor (SMT). A molecular junction transistor contains at its centre a molecule or a quantum dot (QD) connected to two conducting leads which act as a source (S) and a drain (D). The S-QD-D system is placed on a substrate to which is attached gate. The electrons in S and D can be treated as free electrons with continuous momentum states. The central QD contains discrete energy levels and so the QD electrons are described by localized states. Because of the application of a bias voltage, electrons from S can travel to D through QD giving rise to a tunneling current which can also be controlled by the gate voltage. The tunneling of electrons from S to QD and QD to D and vice versa can be described by a hybridization term. Several transport properties have been studied in SMT systems^16–20^ which show potential for promising applications in nano-devices. There have also been investigations on correlation effects in a SMT system namely, the Coulomb blockade and Kondo effect^21–25^. It has also been observed that the electron–electron (e–e) and local electron–phonon (e–p) interactions play a crucial role on the non-equilibrium quantum transport through SMT structures^26–31^. The effect of e–p interaction on the transport properties in an SMT system has been studied by Chen et al.^30^. They have shown that phonon-assisted conductance is reduced significantly in the presence of e–p coupling. Recently, Khedri et al.^31^ have shown the phononic responses in the bias-voltage-dependent electric currents in a vibrating molecular transistor. The effect of quantum dissipation on the tunneling conductance of an SMT system has been investigated by Raju and Chatterjee (RC)^32^. They have assumed that QD contains a single localized lattice mode which interacts with the QD electrons through a coupling of Holstein type. They have further assumed that the insulating substrate contains a large number of uncoupled harmonic oscillator modes and thus acts as a phonon-reservoir. In the RC picture, the substrate phonons can interact with the local QD phonon through the linear Caldeira-Leggett (CL) interaction giving rise to dissipation. They have formulated the whole system by Anderson-Holstein-Caldeira-Leggett (AHCL) model and used the Keldysh non-equilibrium Green function (NEGF) technique to calculate the tunneling current and differential conductance. It has been shown that dissipation renormalizes the QD phonon frequency and consequently the polaronic effect decreases leading to an increase in the tunneling current. Later, Kalla et al.^33^ have studied the transport properties of the same set-up in the presence of an external magnetic field. This work has useful applications for a spin-filtering device. The SO interaction (SOI) is another important characteristic feature that can lead to spin-dependent transport^34–44^. Sun et al.^44^ have given a derivation of the Rashba SO (RSO) interaction in second quantized notation and have shown how RSO interaction (RSOI) and magnetic flux together can polarize the transport properties of a QD in an Aharonov-Bohm ring. Some experimental studies^45,46^ have shown that temperature can also play a significant role on the non-equilibrium transport. Kalla et al. have theoretically analysed the effect of temperature between the source and the drain in an SMT system^47^. Very recently, Kalla et al. have studied the transient dynamics in a dissipative SMT with e–p and e–e interaction^48^.

**Figure 1:**
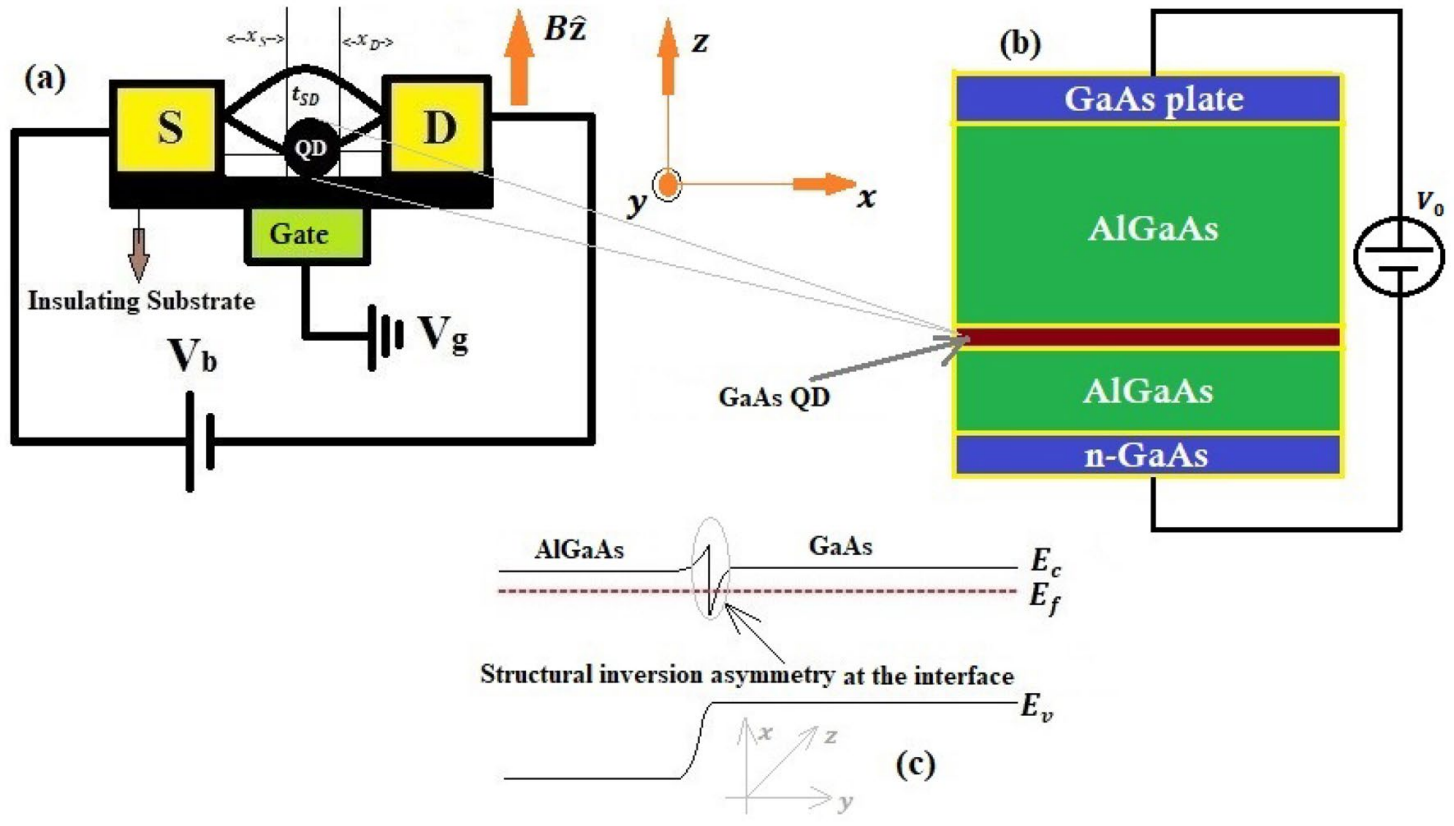
Schematic diagram (a) of a QDT device with a QD containing RSOI embedded in a two-arm loop; (b) for experimental realization of a QD; (c) showing structural inversion asymmetry at the GaAs-AlGaAs interface.

In this study, we wish to investigate the effect of RSOI on the non-equilibrium quantum transport in a dissipative QD transistor (QDT) device. We consider a QDT system in which a two-arm quantum loop containing a QD in one of its arms is sandwiched between the source and the drain (Fig. 1a). Thus, the electrons from S can tunnel to D following two paths, one through the arm of the loop that contains the QD and the other through the arm of the loop that does not contain any QD. We assume that the QD electrons can interact with each other through a Hubbard-like interaction and with the local phonon through an e–p interaction of Holstein type. Following the approach of Sun et al.^44^ we incorporate the RSOI-phase and model the system by AHCL Hamiltonian and employ the finite temperature Keldysh NEGF technique^49^ to calculate the phonon-induced magneto-transport properties in a correlated dissipative QDT structure in the presence of RSOI.

## Theoretical model

The standard model of a QDT with a polar semiconducting QD embedded in a two-arm loop that is attached to two metallic leads namely Source (S) and Drain (D) is depicted in Fig. 1a where the QD placed on one arm of the loop contains RSO, e–p and Hubbard interactions and the other arm (which does not contain RSOI) directly connects S and D with a coupling strength ${t}_{SD}$. A schematic diagram for the realization of the QD used in Fig. 1a is shown in Fig. 1b. It is evident that the heterostructure geometry of Fig. 1b would lead to a band-bending at the GaAs-AlGaAs interfaces giving rise to a structural inversion asymmetry (shown in Fig. 1c) which produces the RSO coupling in the GaAs QD. The red part of Fig. 1b is considered as the central GaAs QD which is attached to S on one side and to D on the other side. A given number of electrons can be accumulated in the QD by using the voltage ${V}_{0}.$ The whole system is mounted on an insulating substrate that contains non-interacting phonons behaving as a phonon-bath which can interact with the QD-phonon giving rise to a quantum damping effect. The bias voltage ${V}_{b}$ and the gate voltage ${V}_{\mathrm{g}}$ are applied as shown in the Fig. 1a. Because of the bias voltage, electrons can travel from S to D by tunnelling through the QD and also by hopping through the other path. It may be noted that the current channel is in the x-direction and a magnetic field ${\varvec{B}}(\mathrm{0,0},B)$ is applied in the z-direction. In general, a QD may have many discrete energy levels, but it may still behave like an SMT system at a sufficiently small size, as the higher energy levels in that case can be disregarded.

The system can be described, in general, by the following AHCL-RSO Hamiltonian

1 $$H = H_{S,D} + H_{QD} + H_{T} + H_{B} ,$$

ere

2 $$H_{S,D} { } = \mathop \sum \limits_{k\sigma \in S,D} \varepsilon_{k} \left( {c_{kS,\sigma }^{\dag } c_{kS,\sigma } + c_{kD,\sigma }^{\dag } c_{kD,\sigma } } \right) + t_{SD } \mathop \sum \limits_{k\sigma \in S,D} \left( {c_{kS,\sigma }^{\dag } c_{kD,\sigma } + h.c.} \right),$$

3 $$\begin{gathered} H_{{{\text{QD}}}} = \mathop \sum \limits_{d\sigma } \left( {\varepsilon_{d} - eV_{\mathrm{g}} - \frac{1}{2}g^{*} \mu_{B} B\sigma_{z} } \right)n_{d\sigma } + \mathop \sum \limits_{d} Un_{d \uparrow } n_{d, \downarrow } \hfill \\ \quad \quad \quad \;\; + \left( {\frac{{p_{0}^{2} }}{{2m_{0} }} + \frac{1}{2}m_{0} \omega_{0}^{2} x_{0}^{2} } \right) + g\mathop \sum \limits_{d\sigma } n_{d\sigma } x_{0} + H_{R} , \hfill \\ \end{gathered}$$

4 $$H_{T} = \mathop \sum \limits_{kd\sigma } \left[ {V_{k} \left( {c_{kS,\sigma }^{\dag } c_{d\sigma } + c_{kD,\sigma }^{\dag } c_{d\sigma } } \right) + h.c} \right],$$

5 $$H_{B} = H_{BO} + H_{QD - B} \equiv \mathop \sum \limits_{i = 1}^{N} \left[ {\frac{{p_{i}^{2} }}{{2m_{i} }} + \frac{1}{2}m_{i} \omega_{i}^{2} x_{i}^{2 } } \right] + \mathop \sum \limits_{i = 1}^{N} \beta_{i} x_{i} x_{0} .$$

Equation (2) represents the lead Hamiltonian ${H}_{S,D}.$ The first term of ${H}_{S,D}$ gives the total energy of the conduction electrons in S (D), where $n_{kS\left( D \right),\sigma } \left( { = c_{kS\left( D \right),\sigma }^{\dag } c_{kS\left( D \right),\sigma } } \right)$ denotes the number operator for the S (D) electrons with momentum $k$ and spin $\sigma$ where $\sigma =+1 \mathrm{and} \sigma =-1$ correspond to spin-up (↑) and spin-down (↓) electrons respectively, $c_{kS\left( D \right),\sigma }^{\dag } \left( {c_{kS\left( D \right),\sigma } } \right)$ being the corresponding creation (annihilation) operator and the second term of ${H}_{S,D}$ represents the coupling between the two leads with the hopping strength ${t}_{SD}$. Equation (3) gives the Hamiltonian (${H}_{\mathrm{QD}})$ for the QD which in general can contain many localized energy levels $d$ with energy ${\varepsilon }_{d}.$ The first term of ${H}_{\mathrm{QD}}$ shows that the QD energy is modified by the gate voltage ${V}_{\mathrm{g}}$ and the magnetic field $B\hat{z}$, where $n_{d\sigma } \left( { = c_{d\sigma }^{\dag } c_{d\sigma } } \right)$ denotes the number operator for the QD electrons, $c_{d\sigma }^{\dag } (c_{d\sigma } )$ being the corresponding creation (annihilation) operator of the $d$-th energy level, $\sigma_{z}$ is the z-component of the Pauli matrices ${\varvec{\sigma}}$, $g^{*}$ is the gyromagnetic ratio and $\mu_{B}$ is the Bohr magneton. The second term of $H_{{{\text{QD}}}}$ represents the Hubbard interaction with $U$ as the Coulomb correlation strength. The third term of $H_{{{\text{QD}}}}$ is the Hamiltonian for the local lattice mode of QD, where $\left( {x_{0} , p_{0} } \right)$ are the coordinate and the corresponding canonical momentum of the QD oscillator with mass $m_{0}$ and frequency $\omega_{0}$ which are respectively given by $x_{0} = \sqrt {\frac{\hbar }{{2m_{0} \omega_{0} }}} \left( {b^{\dag } + b} \right)$ and $p_{0} = i\sqrt {\frac{{\hbar m_{0} \omega_{0} }}{2}} \left( {b^{\dag } - b} \right)$. The fourth term of $H_{{{\text{QD}}}}$ represents the interaction of the QD electrons with the local QD phonon with $g$ giving the strength of the coupling. The fifth term of ${H}_{\mathrm{QD}}$ represents the RSOI which, in general, can be written in the $x-z$ plane as

6 $$H_{R} = \hat{y}.\frac{{\alpha_{R} }}{\hbar }\left[ {{\varvec{\sigma}} \times \left( {{\varvec{p}} + \frac{{e{\varvec{A}}}}{c}} \right)} \right],$$

where ${\alpha }_{R}$ is the strength of RSOI. Choosing the Landau gauge: ${\varvec{A}}=(0,Bx,0)$, we can write ${H}_{R}$ in the second quantized notation in the chosen basis $$|d\sigma \rangle \equiv {\varphi }_{d}({\varvec{r}})\left(\begin{array}{c}1\\ 0\end{array}\right)$$ as

7 $$H_{R} = \frac{{\alpha_{R} }}{\hbar }\mathop \sum \limits_{{dd^{^{\prime}} }} \left[ {t_{{d^{\prime}d}}^{x} \left( {c_{{d^{\prime}\sigma }}^{\dag } c_{d\sigma } - c_{{d^{\prime}, - \sigma }}^{\dag } c_{d, - \sigma } } \right) + t_{{d^{\prime}d}}^{z} \left( {c_{{d^{\prime}, - \sigma }}^{\dag } c_{d\sigma } - c_{d, - \sigma }^{\dag } c_{{d^{\prime}\sigma }} } \right)} \right] + h.c.,$$

where ${t}_{{d}^{^{\prime}}d}^{x(z)}=\int d{\varvec{r}}\boldsymbol{ }{\varphi }_{{d}^{^{\prime}}}^{*}\left({\varvec{r}}\right)\boldsymbol{ }{p}_{x(z)} {\varphi }_{d}({\varvec{r}})$. The first term of Eq. (7) denotes the inter-level hopping between the same spin state and the second term denotes the same between the spin-flip states. Equation (4) represents the tunneling Hamiltonian ${H}_{T}$ which describes the tunneling of electrons from S to D through the QD and that of the reverse process, ${V}_{k}$ being the hybridization strength. Equation (5) represents the substrate Hamiltonian ${H}_{B}$ which contains two pieces, $H_{BO} \;{\text{and}}\;H_{QD - B}$. $H_{BO}$ describes a collection of $N$ uncoupled bath oscillators where $\left({x}_{i}, {p}_{i}\right)$ refer to the generalized coordinates and momenta of the $i$-th bath oscillator of mass ${m}_{i}$ and frequency ${\omega }_{i}$ and ${H}_{QD-B}$ gives the linear interaction between the QD-phonon and the $i$-th bath-phonon with the coupling strength ${\beta }_{i}$. ${H}_{QD-B}$ is chosen in the spirit of the Caldeira-Leggett model^50^.

## Results

We study the RSOI-induced transport properties using the temperature-dependent Keldysh NEGF technique. In particular, we calculate the spin-resolved tunneling current, conductance and spin-polriztion in the presence of e–p interaction, Coulomb interaction and quantum dissipation. In this section, we show the behaviour of these quantities as a function of a few tunable parameters. We normalize the energy scale of the system by the phonon-energy, $\hbar \omega_{0}$. For convenience, we set $\Gamma = 0.2,$ $e{\it{\text{V}}}_{\mathrm{g}} = 0, m^{*} =$ 0.036 $m_{e}$, $eV_{m} = 0.1$, $U = 5$ and $\varepsilon_{d} =$ 0.

### Tunneling current

**Figure 2:**
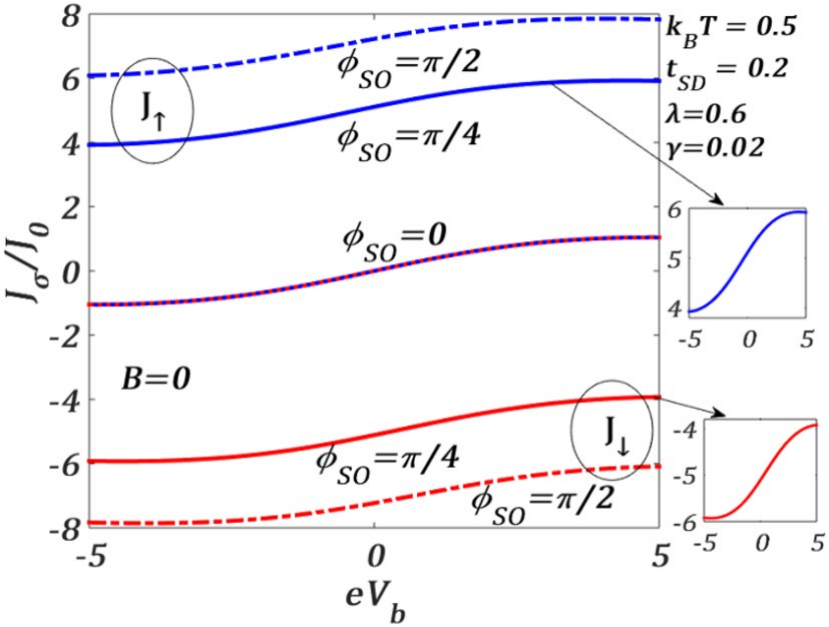
Spin-resolved current ${J}_{\sigma }/{J}_{0}$ versus ${eV}_{b}$ for different values of ${\phi }_{SO}$ for ${k}_{B}T=0.5$, $\lambda =0.6,{t}_{SD}=0.2,\gamma =0.02$ at $B=0$.

In Fig. 2, we present the variation of the spin-resolved normalized tunneling current ${J}_{\sigma }$ (see Eq. 39 in "Methods") at finite temperature $T$ as a function of the bias voltage ${V}_{b}$ for a given set of QDT parameters and different RSOI strength ${\phi }_{SO}$ which is related to ${\alpha }_{R}$ as

8 $$\phi_{SO} = \alpha_{R} \frac{{m^{*} }}{{\hbar^{2} }}l,$$

where $l=\left({x}_{S}-{x}_{D}\right)$ being the length scale over which ${\alpha }_{R}$ is non-zero. ${J}_{\sigma }$ is measured in the units of ${J}_{0}=e/2h$. One can observe that ${J}_{\sigma }$ initially increases with increasing ${V}_{b}$ in a nonlinear way, then shows an Ohmic nature in the middle region and finally saturates after a certain value of ${V}_{b}$. This can be explained as follows. On application of ${\mathrm{\it{V}}}_{\mathrm{\it {b}}}$, the Fermi level of S shifts up and that of the right lead goes down. This causes electrons to enter from the S-lead into QD giving rise to a nonzero tunneling current. But as the QD is able to accommodate only a limited number of electrons, the current gets saturated if ${V}_{b}$ is raised beyond a certain value. One may notice that the tunneling is not significant unless ${V}_{b}$ is high enough. As mentioned above, for a non-zero ${V}_{b}$, S- and D-Fermi levels shift respectively up and down equally and electrons from S-Fermi level jump into the spin-up (spin-down) level of the QD and then go to the D-Fermi level causing a non-zero spin-up (spin-down) current. So, a substantial strength of the bias voltage is required for this tunnelling to happen. However, the more interesting phenomenon here is the splitting of ${J}_{\uparrow}$ and ${J}_{\downarrow}$ for a nonzero value of ${\phi }_{SO}$ even at $B=0$. At ${\phi }_{SO}\ne 0$, the spin degeneracy is removed due to the RSOI and the single degenerate QD energy level splits into spin-up and spin-down levels leading to the separation of the spin-up and spin-down currents ${J}_{\uparrow }$ and ${J}_{\downarrow }$. As this separation between ${J}_{\uparrow }$ and ${J}_{\downarrow }$ is entirely due to RSOI, the graphs for ${J}_{\uparrow }$ and ${J}_{\downarrow }$ obviously merge with each other for ${\phi }_{SO}=0$ in the absence of $B$.

**Figure 3:**
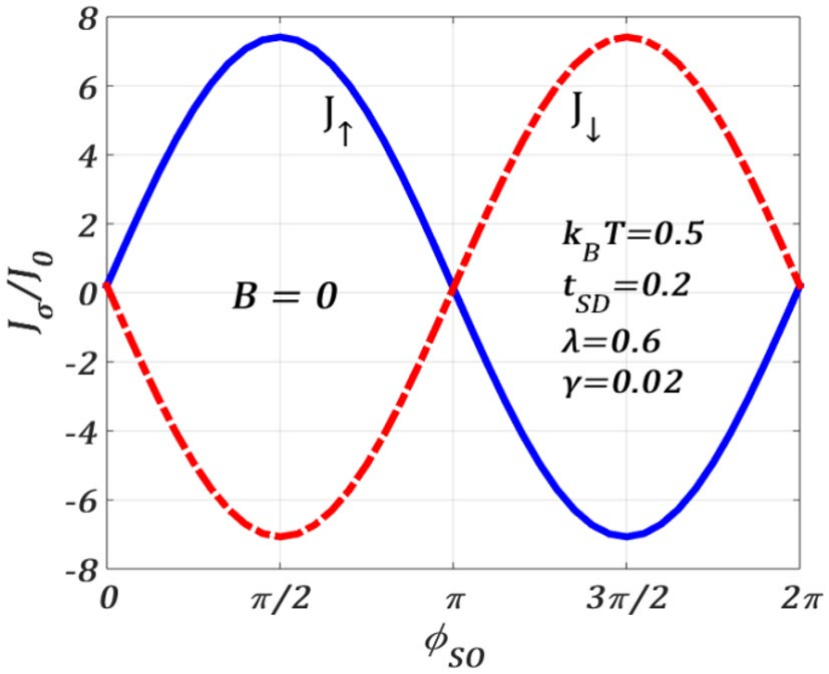
Spin-resolved current ${J}_{\sigma }/{J}_{0}$ versus ${\phi }_{SO}$ for ${k}_{B}T=0.5$, $\lambda =0.6,{t}_{SD}=0.2,\gamma =0.02, {eV}_{b}=0.5$ at $B=0$.

To study the SOI-induced splitting more specifically, we plot ${J}_{\uparrow }$ and ${J}_{\downarrow }$, in Fig. 3, as a function of ${\phi }_{SO}$ at $B=0$ and $T\ne 0$. The periodic behaviour with a period $2\pi$ is clearly visible. At ${\phi }_{SO}=0,$ ${J}_{\uparrow }$ is zero and as ${\phi }_{SO}$ increases, ${J}_{\uparrow }$ also increases and exhibits a maximum at ${\phi }_{SO}=\pi /2$, and then it continues to decrease with further increase in ${\phi }_{SO}$ and shows a minimum at ${\phi }_{SO}=3\pi /2$ after which it again rises and becomes zero at ${\phi }_{SO}=2\pi$. Though both ${J}_{\uparrow }$ and ${J}_{\downarrow }$ have the same period $2\pi ,$ they have the opposite phase. This gives an interesting crossing behaviour in the ${J}_{\uparrow }$ and ${J}_{\downarrow }$—curves. The crossing occurs at those values of ${\phi }_{SO}$ that are even multiples of $\pi /2.$ Obviously, the phase difference between ${J}_{\uparrow }$ and ${J}_{\downarrow }$ in the case of $B=0,$ is caused entirely due to the RSOI. It is important to mention that the spin gap $({J}_{\uparrow }-{J}_{\downarrow })$ can be controlled by varying the RSOI parameter ${\alpha }_{R}$ which can be accomplished by tuning the gate voltage. The spin gap shows maxima at odd-integral multiple values of ${\phi }_{SO}=\pi/2$ and vanishes at even integral values of ${\phi }_{SO}=\pi/2$ including zero.

**Figure 4:**
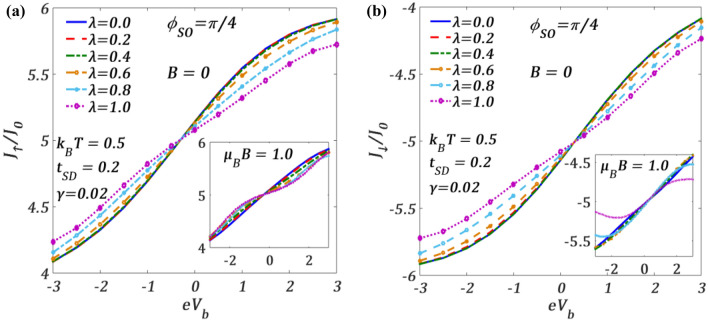
(**a**) ${J}_{\uparrow }/{J}_{0}$ and (**b**) ${J}_{\downarrow }/{J}_{0}$ versus ${eV}_{b}$ for different values of $\lambda$ at a fixed ${\phi }_{SO}$ for ${k}_{B}T=0.5$ and $B=0$. Insets at $\mu_{B} \,B = 1.0$.

In Fig. 4, we plot ${J}_{\uparrow }$ and ${J}_{\downarrow }$ with respect to ${V}_{b}$ for different values of e–p interaction strength $\lambda$ defined as $\lambda =g{\left(1/2{m}_{0}\hslash {\omega }_{0}{{\widetilde{\omega }}_{0}}^{2}\right)}^{1/2}$, at a finite $T$ to see the effect of e–p interaction on ${J}_{\uparrow }$ and ${J}_{\downarrow }$ in the presence of RSOI. Figure 4a shows the behaviour of ${J}_{\uparrow }$ while Fig. 4b presents the behaviour of ${J}_{\downarrow }$. One may notice that for a given ${\phi }_{SO}$, the qualitative behaviour of ${J}_{\uparrow }$ and ${J}_{\downarrow }$ is similar at $B=0$. Both ${J}_{\uparrow }$ and ${J}_{\downarrow }$ decrease with increasing $\lambda$ for positive ${V}_{b}$. This can be understood from the mechanism of polaron formation which impedes the flow of the tunneling of conduction electrons. To be more specific, let us consider, Eqs. (26) and (41) (in “Methods”) which show that the phonon-induced QD-lead hybridization strength ${\widetilde{V}}_{k}$ and QD-lead coupling $\it\Gamma$ (Eq. 40) decrease by the Holstein reduction factor. The Green functions (Eq. 43) and the spectral function (Eq. 53) are also decreased by the polaronic interaction ($\lambda$) and consequently the tunnelling current (see Eq. 39) decreases as polaronic interaction increases. It is also clear from the figure and also from Eq. (41) that for small values of $\lambda$ ($\lambda \lesssim 0.4$), the polaronic effect is marginal. In the insets we show the variations at $\mu_{B} \,B = 1.0$. These figures show that the qualitative variations of ${J}_{\uparrow }$ and ${J}_{\downarrow }$ at a finite value of the magnetic field are different, particularly for higher values of $\lambda$. This implies that, in the presence of a magnetic field, the effect of RSOI on ${J}_{\uparrow }$ and ${J}_{\downarrow }$ is qualitatively different. This can be explained from Eq. (23) (see “Methods”), which shows that the effective dot-energy ${\widetilde{\varepsilon }}_{d\sigma }$ is different for spin-up and spin-down electrons. The expression of ${\widetilde{\varepsilon }}_{d\sigma }$ also shows that for the spin-down electrons, there exists a competition between the polaronic energy and the magnetic energy, whereas no such competition exists for the spin-up electrons. One may also observe that the changes in current densities in the presence of magnetic field for lower values of $\lambda$ are minimal for the chosen set of parameters.

**Figure 5:**
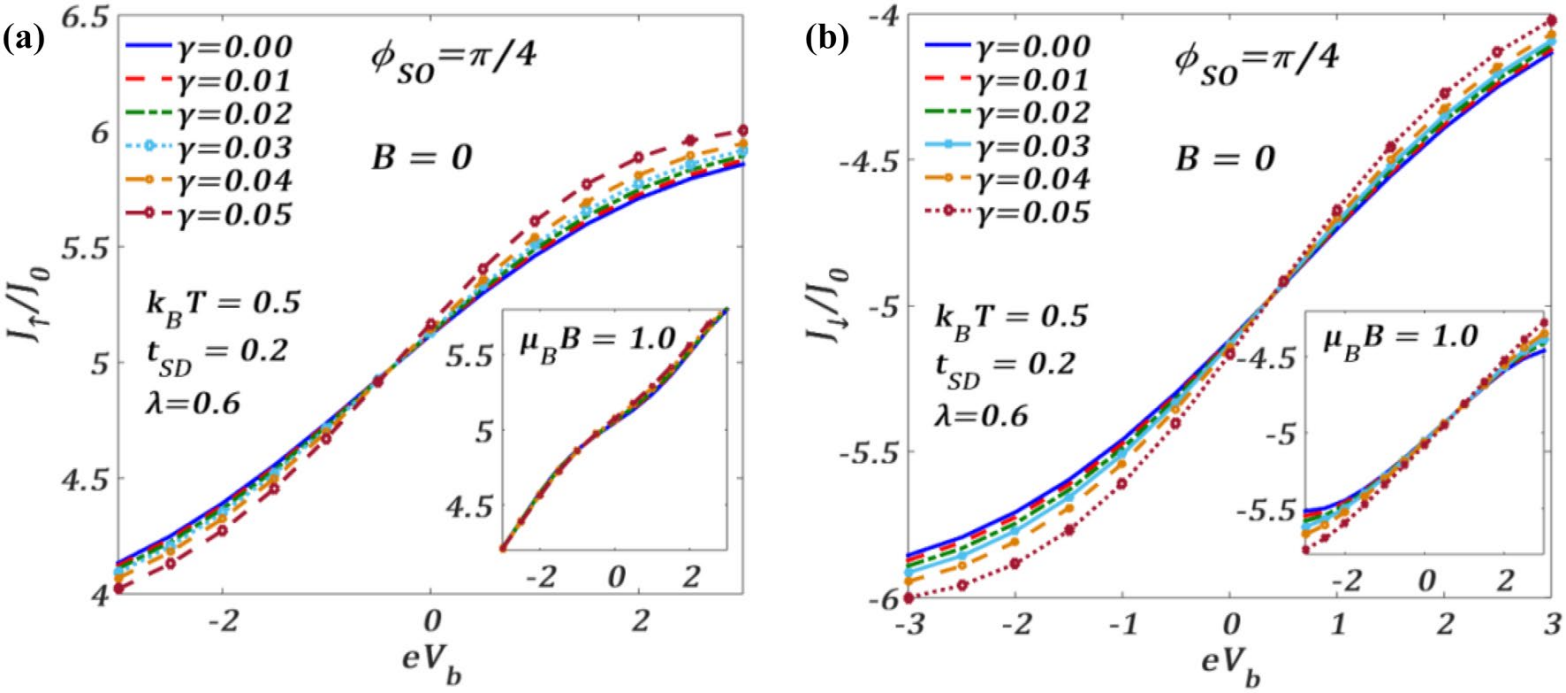
(**a**) ${J}_{\uparrow }/{J}_{0}$ and (**b**) ${J}_{\downarrow }/{J}_{0}$ versus ${eV}_{b}$ for different values of $\gamma$ at a fixed ${\phi }_{SO}$ for ${k}_{B}T=0.5$ and $B=0$. Insets at $\mu_{B} \,B = 1.0$.

Figure 5 describes the effect of quantum dissipation (parameterized by $\gamma$) on spin current densities in the presence of ${\phi }_{SO}$ at a finite value of $T$. It is evident that for positive ${V}_{b}$, ${J}_{\uparrow }$ and ${J}_{\downarrow }$ increase as $\gamma$ increases. This can be explained as follows. The coupling of the bath phonons with the QD phonon reduces the frequency of the phonon ${\omega }_{0}$ to ${\widetilde{\omega }}_{0}$ which apparently means that the QD lattice mode undergoes a frictional effect which is precisely the effect of dissipation. This effect reduces the e–p interaction and consequently increases the tunneling current. Here, again the insets suggest that at finite $B$, the variations of ${J}_{\uparrow }$ and ${J}_{\downarrow }$ with $\gamma$ are different, though $\gamma$ enhances both ${J}_{\uparrow }$ and ${J}_{\downarrow }$. At $B\ne 0$, the variations of ${J}_{\downarrow }$ are much more prominent than those of ${J}_{\uparrow }$.

**Figure 6:**
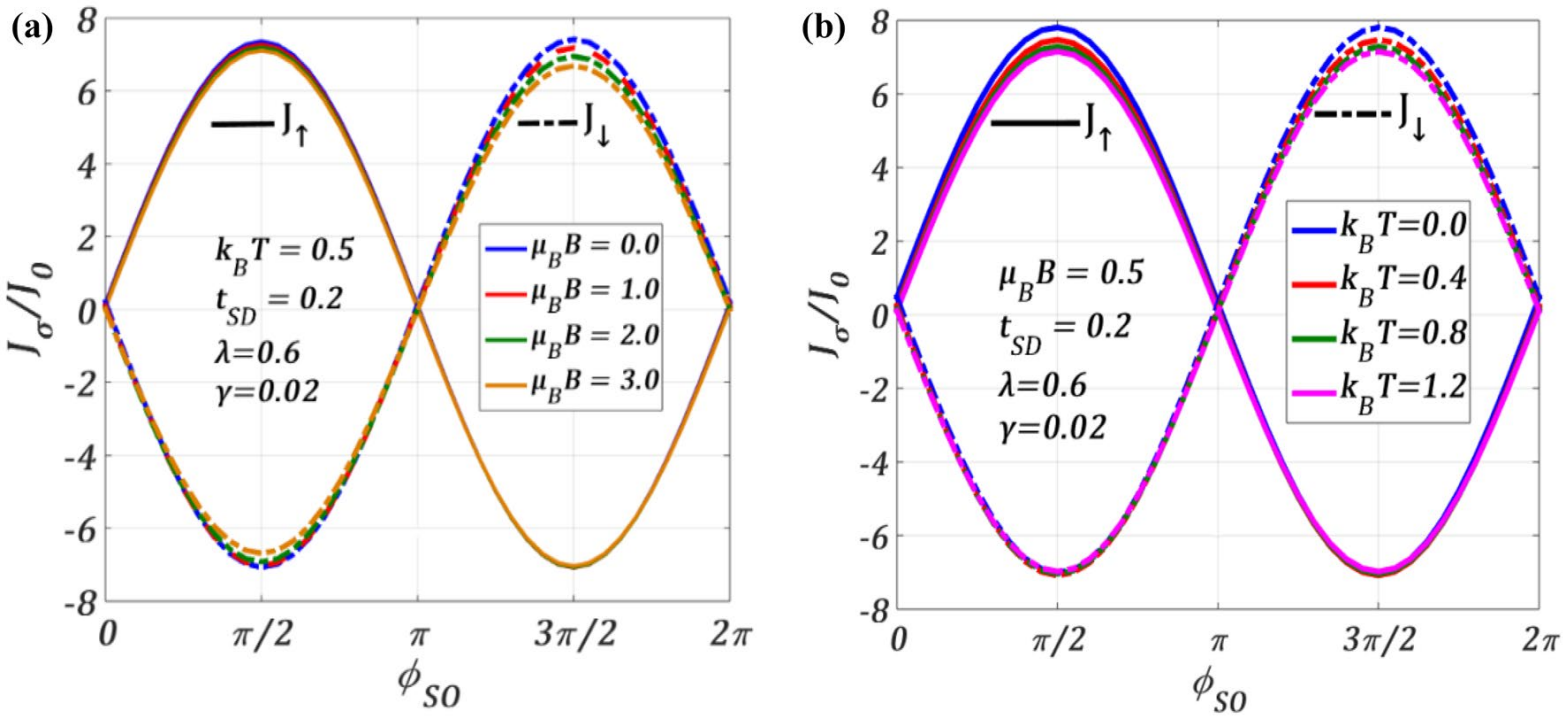
Spin-resolved current ${J}_{\sigma }/{J}_{0}$ versus ${\phi }_{SO}$ at $\lambda =0.6, {t}_{SD}=0.2, \gamma =0.02, { eV}_{b}=0.5$ for different values of (**a**) $B$ at ${k}_{B}T=0.5;$ (**b**) $T$ at $\mu_{B} \,B = 0.5$.

In Fig. 6, we study how ${J}_{\sigma }$ changes with ${\phi }_{SO}$ at different values of the magnetic field and temperature in a particular window of the QDT parameters. In Fig. 6a, we present the effect of the magnetic field and in Fig. 6b the effect of temperature. We observe that, in general, ${J}_{\sigma }$ reduces with the increase in both $T$ and $B$. From Fig. 6a, we see that though the change in ${J}_{\uparrow }$ with $B$ is only marginal, ${J}_{\downarrow }$ exhibits a visible change with $B,$ especially for higher values of SO coupling (for $\pi \le {\phi }_{SO}\le 2\pi$)*.* This again suggests that because of the magnetic field, SOI effects in ${J}_{\uparrow }$ and ${J}_{\downarrow }$ become different. Mathematical analysis (see Eq. 39 in “Methods”) shows that the change in ${J}_{\sigma }$ is mostly dependent on ${\widetilde{G}}_{dd}^{r(a)}$ and the denominator for ${J}_{\uparrow }$ ($\sigma =+1$) is greater than that of ${J}_{\downarrow }$ ($\sigma =-1$) for a given set of parameters. This makes the gap between the ${J}_{\downarrow }$-curves for two values of $B$ larger than that of the corresponding ${J}_{\uparrow }$ curves. Thus, the localizing effect of $B$ is stronger in the case of ${J}_{\downarrow }$ than in the case of ${J}_{\uparrow }$. We can explain the reduction in the current densities with increasing $B$ in the following way. The presence of $B$ gives rise to an additional spitting of the QD’s energy level, the spin-down level rising up and the spin-up level shifting down. As $\mathrm{\it{B}}$ increases, the splitting also increases and for a given ${\phi }_{SO}$, it may so happen that the rise in the spin-down level becomes more than the downshift in the spin-up level. This can cause a large mismatch between the S-Fermi level of the source and the spin-down of the QD giving rising to a lesser probability of S-electrons to tunnel and consequently ${J}_{\downarrow }$ decreases with increasing field. Figure 6b shows the variation of ${J}_{\sigma }$ with $T$. As the phonon excitations increase with increasing $T$, ${J}_{\sigma }$ reduces as $T$ increases, but unlike in the case of Fig. 6a, here ${J}_{\uparrow }$ and ${J}_{\downarrow }$ will be affected equally at a particular temperature. Interestingly, the change in ${J}_{\uparrow }$ and ${J}_{\downarrow }$ by increasing $T$ is significant in the regimes $0\le {\phi }_{SO}\le \pi$ and $\pi \le {\phi }_{SO}\le 2\pi$ respectively. This can be explained as follows. The distribution of the lead-electrons is smeared out by the thermal broadening and the change in ${\mathrm{\it{J}}}_{\uparrow }$ is noticeable if ${\mathrm{\it{k}}}_{\mathrm{\it{B}}}\mathrm{\it{T}}\gtrsim {\Delta }_{\mathrm{R}}(\propto {\mathrm{\alpha}}_{\mathrm{\it{R}}})$, where ${\Delta }_{\mathrm{R}}$ is the spin gap in the QD. This is true for the region: $0\le {\phi }_{\mathrm{SO}}\le\pi$. However, as ${\phi }_{\mathrm{SO}}$ increases, the change in ${\mathrm{\it{J}}}_{\uparrow }$ due to $\mathrm{\it{T}}$ becomes marginal especially for ${\mathrm{\it{k}}}_{\mathrm{\it{B}}}\mathrm{\it{T}}\ll {\Delta }_{\mathrm{R}}$, because in this case, splitting becomes large and the thermal change in the energy of the metallic electrons becomes unimportant for the spin-up electrons. Consequently ${\mathrm{\it{J}}}_{\uparrow }$ does not change in the region: $\pi \le {\phi }_{\mathrm{SO}}\le 2\pi$, as $\mathrm{\it{T}}$ increases. The situation is completely opposite in ${\mathrm{\it{J}}}_{\downarrow }$-case. The first two terms of Eq. (39) show this competition between $\mathrm{\it{T}}$ and ${\phi }_{\mathrm{SO}}$.

**Figure 7:**
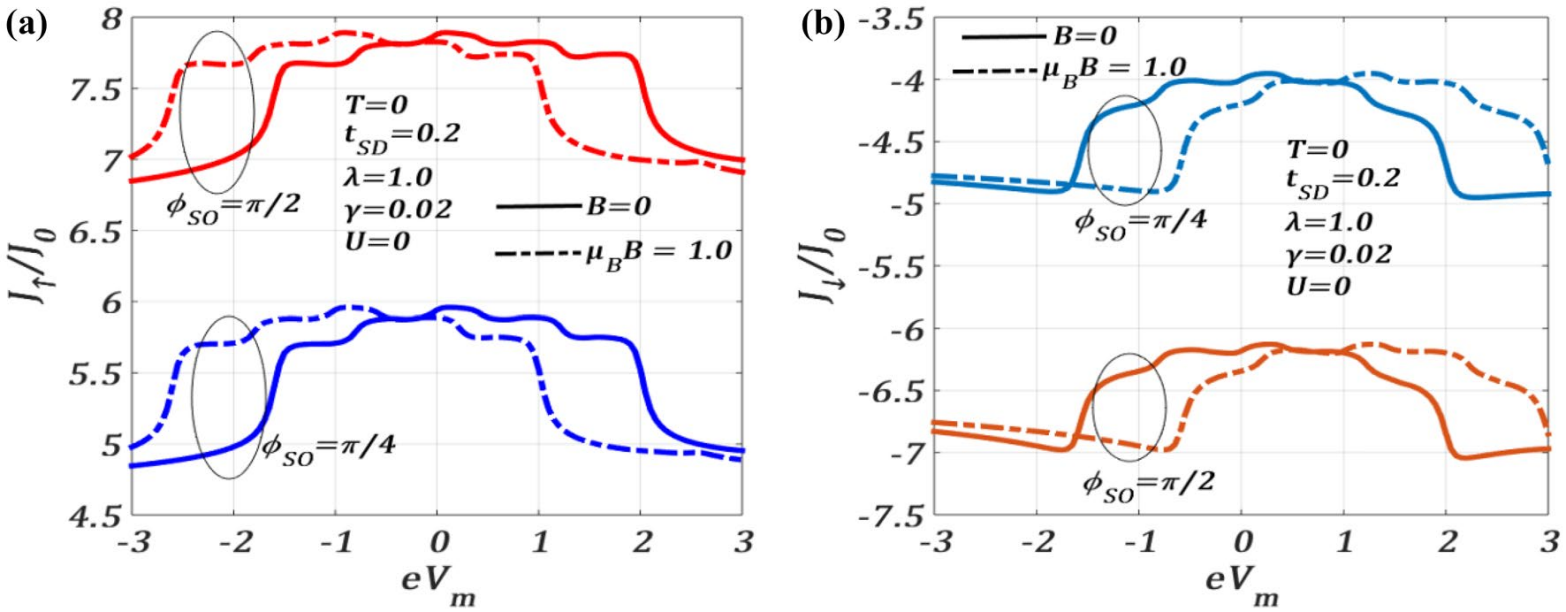
(**a**) ${J}_{\uparrow }/{J}_{0}$ and (**b**) ${J}_{\downarrow }/{J}_{0}$ versus ${eV}_{m}$ for different ${\phi }_{SO}$ at $T=0$, $\lambda =1.0,U=0,\gamma =0.02,$ ${eV}_{b}=3.6$ for $B=0$ and $B\ne 0$.

In Fig. 7, we study the variation of ${J}_{\sigma }$ both for $B=0$ and $B\ne 0$ at $T=0$ with respect to the mid-voltage ${V}_{m}$ which is the average of the potentials of the two leads defined as $e{V}_{m}=\left({\mu }_{S}+{\mu }_{D}\right)/2,$ where ${\mu }_{S}$ and ${\mu }_{D}$ are the corresponding chemical potentials of S and D respectively. One can notice that ${J}_{\sigma }$ exhibits multiple plateaus and shows a maximum around ${V}_{b}=0$. Chen et al.^30^ have studied this variation at zero temperature for $\lambda =0$ and $\lambda =1$ in the absence of a magnetic field, Coulomb correlation, SOI and dissipation and have obtained plateaus in the current density for $\lambda =1$. We observe similar plateaus in the presence of SOI and dissipation, although the value of the current density is much larger in our case. The figures also suggest that the current at ${\phi }_{SO}=\pi /2$ is larger than that at ${\phi }_{SO}=\pi /4$. Interestingly, at non-zero $B,$ ${J}_{\uparrow }$ undergoes a rigid shift towards left on the ${V}_{m}$ axis while ${J}_{\downarrow }$ shifts towards right.

### Differential conductance

In this section, we numerically calculate the differential conductance, ${G}_{\sigma }$ (see Eq. 66) in the presence of e–p interaction, Coulomb correlation and quantum dissipation. The conductance is calculated in units of ${G}_{0}={e}^{2}/2h$.

**Figure 8:**
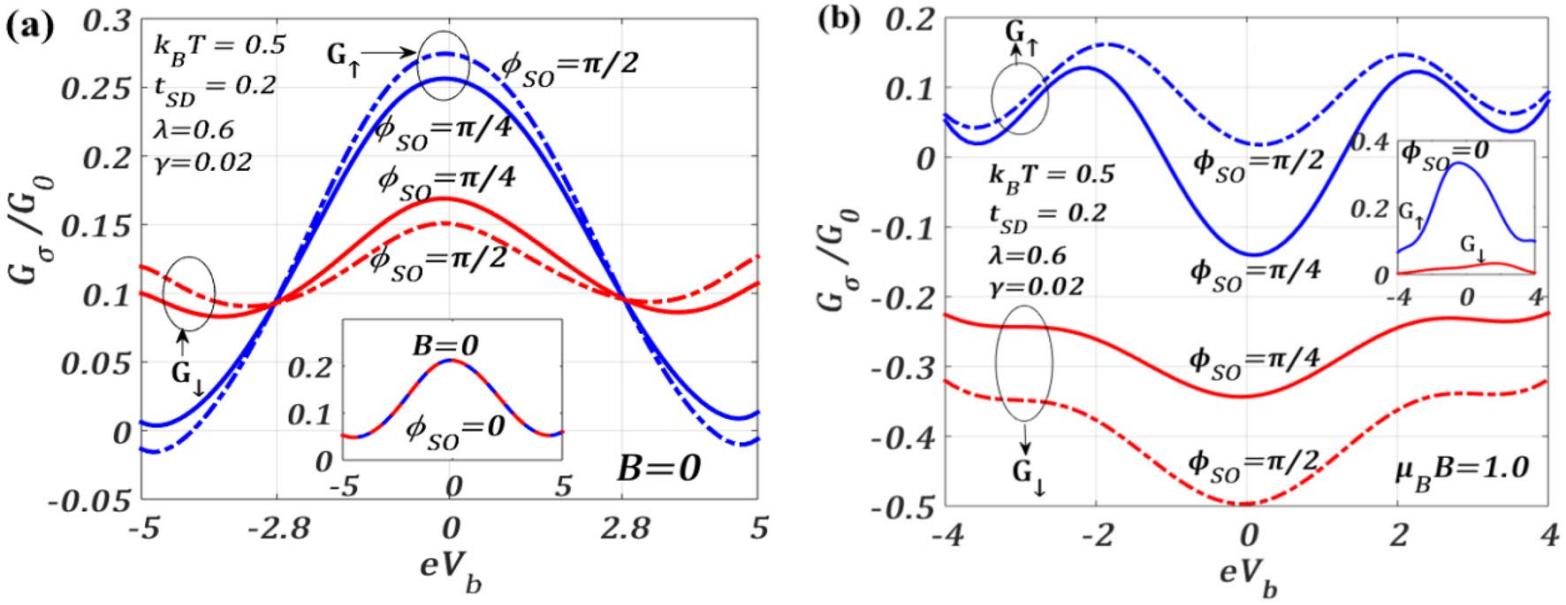
Spin-resolved differential conductance ${G}_{\sigma }/{G}_{0}$ versus ${eV}_{b}$ for different values of ${\phi }_{SO}$ at ${k}_{B}T=0.5$ (**a**) $B=0$, (**b**) $\mu_{B} \,B = 1.0$.

We investigate in Fig. 8, the bahaviour of the spin-resolved differential conductance ${G}_{\sigma }$ as a function of the bias voltage ${V}_{b}$ for different values of ${\phi }_{SO}$ and a set of QDT parameters both in the absence and presence of a magnetic field $B$. Figure 8a provides the results for $B=0$ while Fig. 8b gives the results for $B\ne 0.$ Fig. 8a shows that variation of ${G}_{\sigma }$ with ${V}_{b}$ is Gaussian-like with a maximum (${G}_{\sigma ,max}$) at ${V}_{b}=0.$ The variation is also symmetric with respect to ${V}_{b}=0$. As expected, ${G}_{\sigma }$ splits into ${G}_{\uparrow }$ and ${G}_{\downarrow }$ as we switch on ${\phi }_{SO}$ at $B=0$. The solid lines describe the variations for ${\phi }_{SO}=\pi /4$ and the dotted lines for ${\phi }_{SO}=\pi /2$. The peak height of ${G}_{\uparrow }$ is greater than that of ${G}_{\downarrow }$. It can be seen that for $\left|{V}_{b}\right|<$ 2.8, ${G}_{\uparrow }$(${G}_{\downarrow }$) is larger (smaller) for ${\phi }_{SO}=\pi /2$ than for ${\phi }_{SO}=\pi /4$, but for $\left|{V}_{b}\right|>$ 2.8, the situation reverses. ${G}_{\uparrow }$ and ${G}_{\downarrow }$ cross each other at ${V}_{b}=\pm$ 2.8. The inset shows no splitting at ${\phi }_{SO}=0$ which implies ${G}_{\uparrow }={G}_{\downarrow }$ in this case. Figure 8b shows that the variations are a little different in the presence of a magnetic field. Interestingly, the graphs now exhibit a central minimum at ${V}_{b}=0$ with two more minima, one on each side of ${V}_{b}=0,$ placed symmetrically at higher value of $\left|{eV}_{b}\right|$. The curves for ${G}_{\uparrow }$ and ${G}_{\downarrow }$ do not cross each other at any value of the bias voltage. It is clearly evident that the gap between the ${G}_{\uparrow }$ and ${G}_{\downarrow }$- curves increase as ${\phi }_{SO}$ is changed from $\pi /4$ to $\pi /2$. The gap between ${\phi }_{SO}=\pi /2$ and ${\phi }_{SO}=\pi /4$ curves also increases in the case of $B\ne 0$. As mentioned earlier, this splitting between ${G}_{\uparrow }$ and ${G}_{\downarrow }$ caused by ${\phi }_{SO}$ can be manipulated by tuning the gate voltage which alters ${\phi }_{SO}\left(\propto {\alpha }_{R}\right).$ The inset shows that in the case of ${\phi }_{SO}=0,$ splitting still occurs due to the magnetic field.

**Figure 9:**
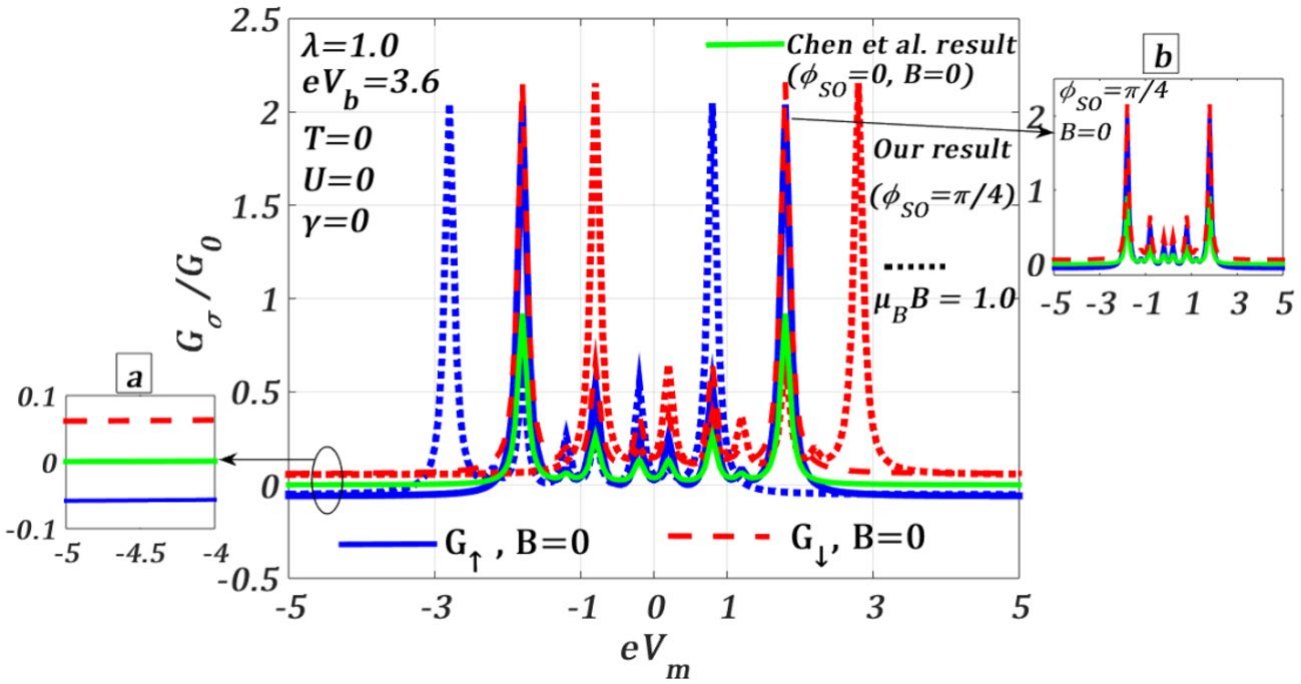
Spin-resolved differential conductance ${G}_{\sigma }/{G}_{0}$ versus ${eV}_{m}$ for ${\phi }_{SO}=\pi /4$ at $T=U=\gamma =0$, $\lambda =1.0,$ ${eV}_{b}=3.6$ for $B=0$ and $B\ne 0$: comparison with the Chen et al.^30^ result.

In Fig. 9, we study the behaviour of the differential conductance ${G}_{\sigma }$ as a function of mid-voltage ${V}_{m}$ in the presence of RSOI ${\phi }_{SO}$ for both $B=0$ and $B\ne 0$ with $\lambda =1.0$. We also compare our results with those of Chen et al.^30^ who have studied the same in the absence of RSOI and magnetic field. They have observed a few satellite peaks in the conductance along with two zero-phonon peaks (taller peaks) symmetrically distributed (solid light green curve at $B={\phi }_{SO}=0$) with respect to ${V}_{m}=0$ and suggested that these satellite peaks occur because of the phonon-assisted tunnelling. We like to see the effects of the RSOI and magnetic field on ${G}_{\sigma }$ for the same parameter values considered by Chen et al. In the presence of RSOI (${\phi }_{SO}=\pi /4$) alone, it can be clearly seen that the solid light green curve splits into two curves (shown in the inset (a)) corresponding to the spin-up (${G}_{\uparrow }$, solid blue) and spin-down (${G}_{\downarrow }$, dashed red) spin-resolved conductances respectively. One can also see that the zero-phonon peaks and the satellite peaks generated by the e–p interaction are symmetric with respect to ${V}_{m}=0$. We would like to mention that the conductance peak heights increase and become sharper in the presence of RSOI, although the zero-phonon up and down-spin peaks merge at a particular ${V}_{m}$. The inset (b) shows that the ${G}_{\downarrow }$- peaks (dashed red) are higher than the ${G}_{\uparrow }$- peaks (solid blue). The enhancement of ${G}_{\sigma }$ by RSOI can be explained as follows. In the presence of RSOI, the current in the QD cannel increases because of an additional interference effect due to the hopping current in the direct channel. This interference effect can be seen from the expression of ${J}_{\sigma }$ (Eq. 39) where sin/cos-terms of SO phase ${\phi }_{SO}$ are modified by the direct channel hopping parameter ${t}_{SD}$*.* As we turn on $B$ ($\mu_{B} \,B = 1.0$) in addition to RSOI, ${G}_{\uparrow }$ undergoes a rigid shift towards left and ${G}_{\downarrow }$ towards right equally and as a result the zero-phonon up-spin (dotted blue curve) and down-spin (dotted red curve) conductance peaks split, though the heights of the peaks remain the same as in the case of $B=0$. Thus, the RSOI enhances the phonon-assisted conductance by increasing the peak heights and the magnetic field splits the peaks. This signature of the peak pattern in spin-resolved conductances can also be understood from Fig. 7, where one can see the boundary lines before and after the plateaus associated with the phonon-mediated conductance peaks. The left–right shift at $B\ne 0$ can also be seen in Fig. 7. Here we have shown results in the absence of quantum dissipation. Similar studies can also be carried out in the presence of dissipation.

**Figure 10:**
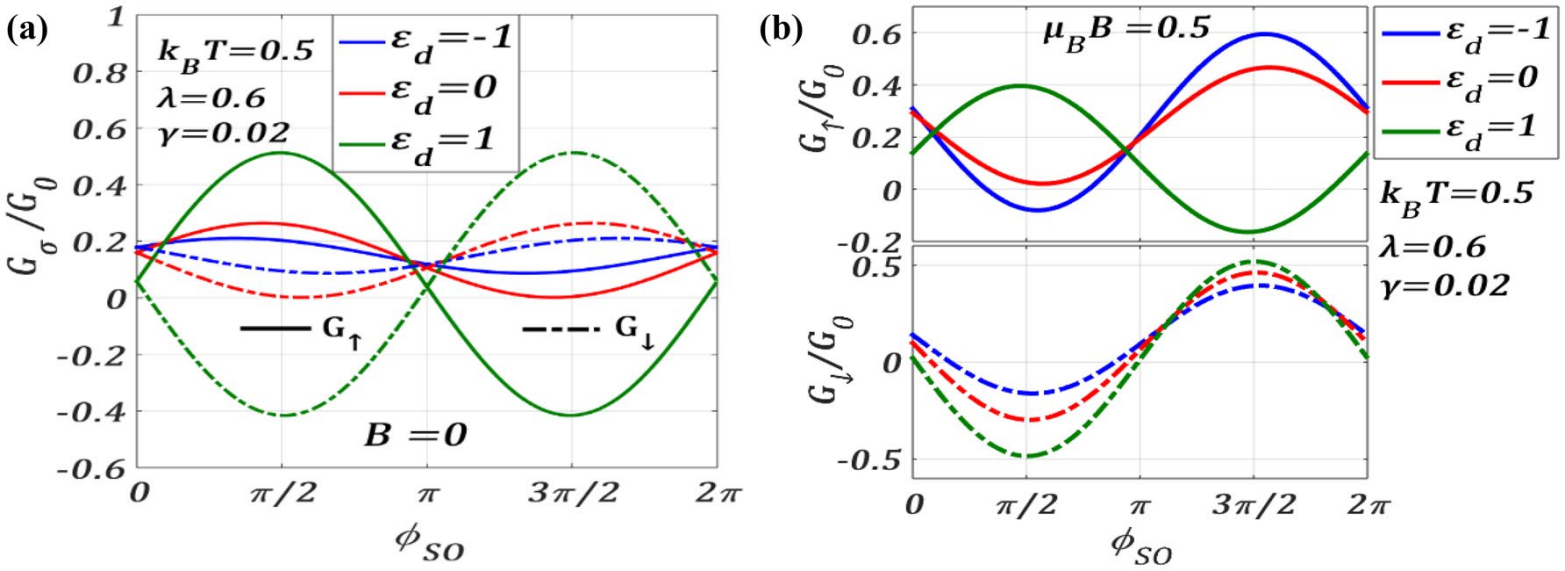
(**a**) Spin-resolved differential conductance ${G}_{\sigma }/{G}_{0}$ versus ${\phi }_{SO}$ for different values of dot energy ${\varepsilon }_{d}$ at $\lambda =0.6,{t}_{SD}=0.2,\gamma =0.02, {eV}_{b}=0.5$ (**a**) for $B=0$ (**b**) $\mu_{B} \,B = 0.5$.

Figure 10 displays the nature of ${G}_{\sigma }$ with respect to ${\phi }_{SO}$ for different values of dot energy. Figure 10a provides results for $B=0$ and Fig. 10b gives results for non-zero values of $B.$ From Fig. 10a, we see that the variation of ${G}_{\sigma }$ with ${\phi }_{SO}$ is $2\pi$-periodic, though ${G}_{\uparrow }$ and ${G}_{\downarrow }$ are out of phase by $\pi$ in conformity with the plots of ${J}_{\sigma }$ vs ${\phi }_{SO}$ shown in Figs. 3 and 6. As the dot energy ${\varepsilon }_{d}$ can be varied by tuning the gate voltage ${\mathrm{\it{V}}}_{\mathrm{g}}$, we consider three values of ${\varepsilon }_{d}$ namely, ${\varepsilon }_{d}=-1,$ ${\varepsilon }_{d}=0$ and ${\varepsilon }_{d}=1$. It is clear from Fig. 10a that as ${\varepsilon }_{d}$ increases, ${G}_{\uparrow }$ increases in the range, 0 $\le {\phi }_{SO}\le \pi$, and decreases in the range, $\pi \le {\phi }_{SO}\le 2\pi$, and shows extrema at ${\phi }_{SO}=p\pi /2$, $p=\mathrm{1,3},5,\dots$ . The behaviour of ${G}_{\downarrow }$ with ${\varepsilon }_{d}$ is just the opposite to that of ${G}_{\uparrow }$ versus ${\varepsilon }_{d}$ and can be obtained from the results of ${G}_{\uparrow }$ by giving a $\pi$ shift. The quantitative difference between the results of ${G}_{\uparrow }$ and ${G}_{\downarrow }$ is particularly significant for positive ${\varepsilon }_{d}$. One can see in Fig. 10b that in the case of $B\ne 0$, ${G}_{\uparrow }$ and ${G}_{\downarrow }$ behave differently from those at $B=0$ and the constant phase correlation between ${G}_{\uparrow }$ and ${G}_{\downarrow }$ is absent except for the case of ${\varepsilon }_{d}=1,$ where again ${G}_{\uparrow }$ and ${G}_{\downarrow }$ as a function of ${\phi }_{SO}$ have a phase difference of $\pi$.

**Figure 11:**
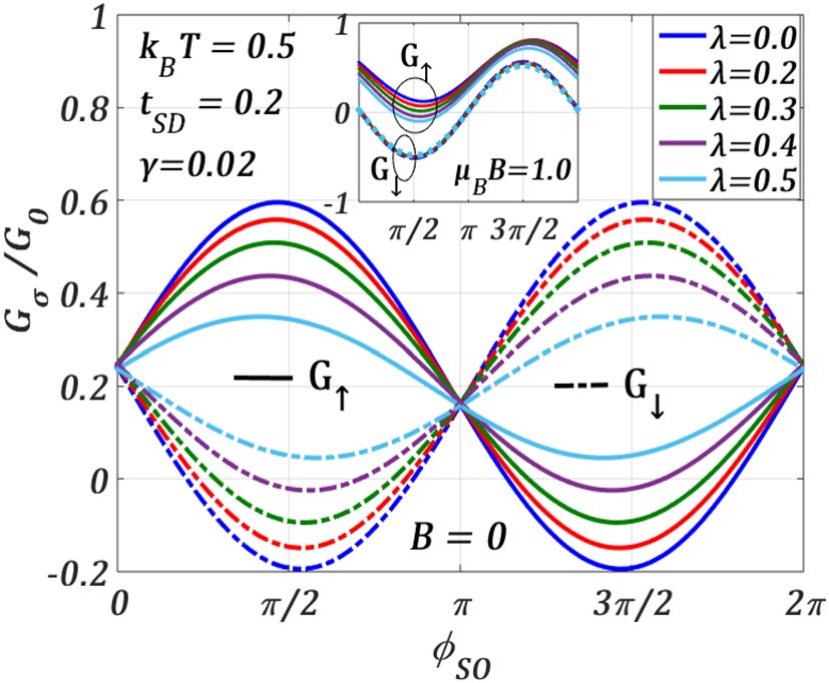
${G}_{\sigma }/{G}_{0}$ versus ${\phi }_{SO}$ for different $\lambda$ values at ${k}_{B}T=0.5$ ${t}_{SD}=0.2,\gamma =0.02, {eV}_{b}=0.5$ for $B=0$. Inset: at $\mu_{B} \,B = 0.5$.

To study the effects of polaronic interaction on spin-resolved conductance ${G}_{\sigma },$ we plot, in Fig. 11, ${G}_{\sigma }$ as a function of ${\phi }_{SO}$ for different values of e–p interaction strength $\lambda$ at $B=0$ for a given set of QDT parameters. As discussed above, ${G}_{\uparrow }$ and ${G}_{\downarrow }$ as a function of ${\phi }_{SO}$ are opposite in phase. For $0\le {\phi }_{SO}\le \pi$, the peak-height of ${G}_{\uparrow }$ decreases with increasing $\lambda$ while that of ${G}_{\downarrow }$ increases. The behaviour becomes just opposite in the region: $\pi \le {\phi }_{SO}\le 2\pi .$ Thus, the e–p interaction which induces the formation of polarons, does not always reduce ${G}_{\sigma },$ rather the effect of e–p interaction also depends on the strength of ${\phi }_{SO}$. This implies that there exists an interesting interplay between the Rashba and e–p interactions that has a significant and decisive effect on the transport process. The inset shows the variations at a finite $B$ where the phase correlation between ${G}_{\uparrow }$ and ${G}_{\downarrow }$ disappears and the variations of ${G}_{\uparrow }$ and ${G}_{\downarrow }$ with respect to ${\phi }_{SO}$ become very different. As a magnetic field is switched on, the maxima and minima in ${G}_{\uparrow }$ as a function of ${\phi }_{SO}$ exchange their positions. Interestingly, at $B\ne 0$, ${G}_{\uparrow }$ always decreases with increasing $\lambda$, though the rate of decrease changes as ${\phi }_{SO}$ increases. However, the variation of ${G}_{\downarrow }$ does not change much for the set of parameters used in this work. This can again be understood from the mathematical analysis given in “Methods” (Eq. 24) where we have shown that in ${\widetilde{\varepsilon }}_{d,-}$, polaronic and magnetic energies are of opposite sign, while ${\widetilde{\varepsilon }}_{d,+}$ is lowered by both the energies. Thus, there exists a competition between the magnetic and polaronic energies in ${J}_{\downarrow }$ which is however absent in ${J}_{\uparrow }$. As a result, ${J}_{\uparrow }$ varies monotonically with $\lambda$ for a given value of $B$, whereas for the same value of $B,$ ${J}_{\downarrow }$ may not change much with $\lambda$ and consequently, the variations of ${G}_{\uparrow }$ and ${G}_{\downarrow }$ become different. We wish to mention that as we turn on the magnetic field, one can see a clear separation between ${G}_{\uparrow }$ and ${G}_{\downarrow }$ curves vertically like the Zeeman splitting for a given ${\phi }_{SO}$ which can, of course, be tuneable. This can also be observed in Figs. 8b and 10b.

**Figure 12:**
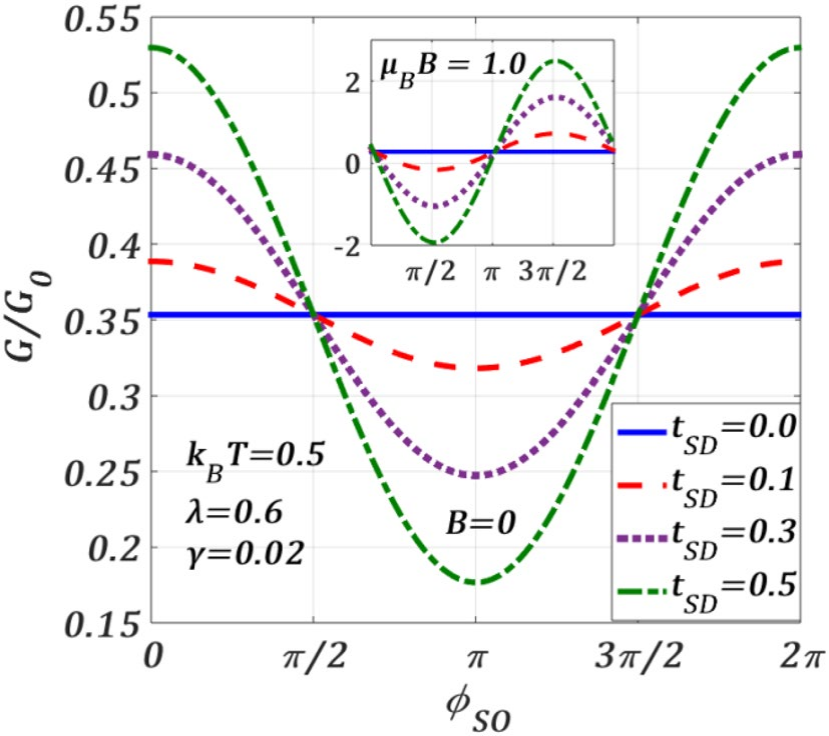
Total $G$ versus ${\phi }_{SO}$ for different ${t}_{SD}$ values at ${k}_{B}T=0.5$ $\lambda =0.6,\gamma =0.02, {eV}_{b}=0.5$ for $B=0$. Inset: at $\mu_{B} \,B = 1.0$.

As the SOI-induced current contains the hopping parameter ${t}_{SD}$, it would be interesting to study the behaviour of the total conductance $G$(= $\sum_{\sigma }d{J}_{\sigma }/d{V}_{b}$) as a function of ${\phi }_{SO}$ for different values of ${t}_{SD }.$ The results are presented in Fig. 12. Let us first describe the results for $B=0.$ The figure shows that for ${t}_{SD}=0$, $G$ is independent of ${\phi }_{SO}.$ At a finite value of ${t}_{SD },$ as ${\phi }_{SO}$ increases from zero, $G$ initially decreases, then forms a minimum at ${\phi }_{SO}=\pi$ and finally increases with the further increase in ${\phi }_{SO}.$ It is clear from the plot that $G$ increases with increasing ${t}_{SD}$ for $0\le {\phi }_{SO}\le \pi /2$ and $3\pi /2\le {\phi }_{SO}\le 2\pi$, while in the window $\pi /2\le {\phi }_{SO}\le 3\pi /2$, it decreases as ${t}_{SD}$ increases. In the inset, we show the variations at $B\ne 0$, where one can notice that $G$ reduces with increasing ${t}_{SD}$ in the region $0\le {\phi }_{SO}\le \pi$, while it decreases with ${t}_{SD}$ in the other half i.e., in the region $\pi \le {\phi }_{SO}\le 2\pi$. Interestingly, for ${t}_{SD }=0$, $G$ remains zero over the entire range of ${\phi }_{SO}$.

**Figure 13:**
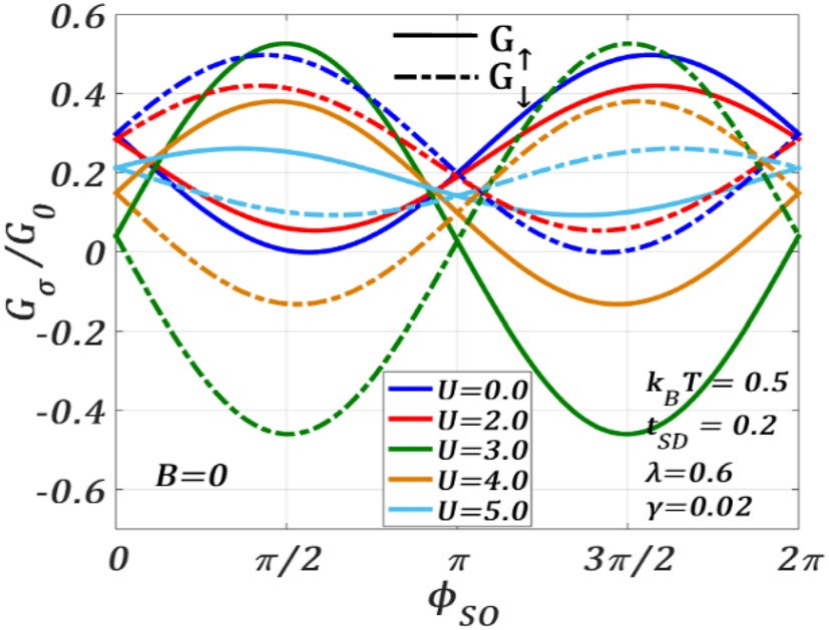
${G}_{\sigma }/{G}_{0}$ versus ${\phi }_{SO}$ for different $U$ values at ${k}_{B}T=0.5$, ${t}_{SD}=0.2,\gamma =0.02,\lambda =0.6, {eV}_{b}=0.5$ for $B=0$.

**Figure 14:**
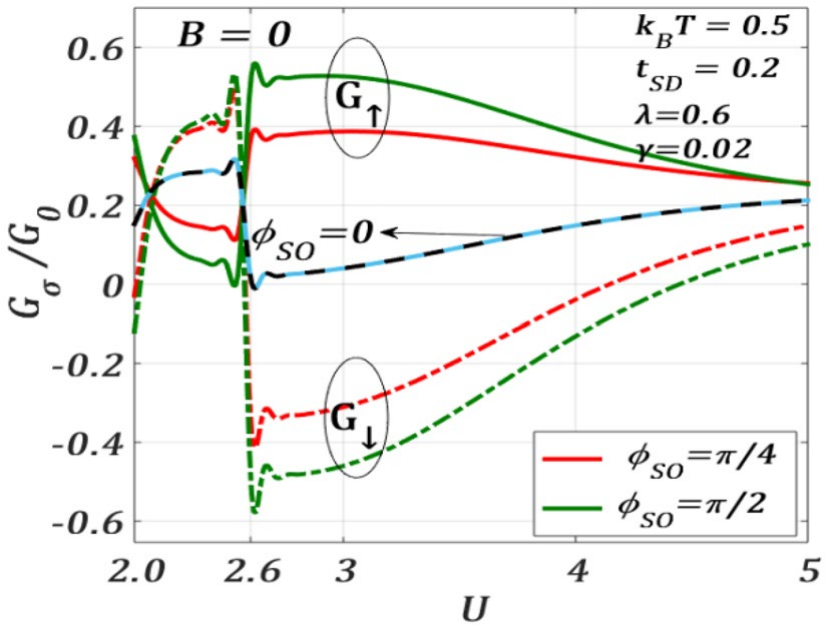
${G}_{\sigma }/{G}_{0}$ versus $U$ for different ${\phi }_{SO}$ values at ${k}_{B}T=0.5$, ${t}_{SD}=0.2,\gamma =0.02,\lambda =0.6, {eV}_{b}=0.5$ for $B=0$.

Figure 13 displays the variation of spin-polarized conductance ${G}_{\sigma }$ with ${\phi }_{SO}$ for different values of $U$ at $B=0$. ${G}_{\sigma }$ exhibits an interesting behaviour with respect to $U$. For $U=0$ and $2$, ${G}_{\uparrow }$ has a minimum at around ${\phi }_{SO}=\pi /2$ and a maximum at around ${\phi }_{SO}=3\pi /2$. However, for $U>2$, ${G}_{\uparrow }$ changes its phase by around $\pi$, showing maximum and minimum at ${\phi }_{SO}=\pi /2$ and ${\phi }_{SO}=3\pi /2$ respectively. It is interesting to see that ${G}_{\downarrow }$ and ${G}_{\uparrow }$ are opposite in phase with respect to ${\phi }_{SO}$ for all values of $U$. Thus, there exists a critical value of $U$ at which the phase of ${G}_{\sigma }$ reverses with respect to ${\phi }_{SO}$. To explore this critical behaviour, we plot ${G}_{\sigma }$ as a function of $U$ for different values of ${\phi }_{SO}$ at $B=0$ in Fig. 14. As our main interest is to locate the transition point, we consider only a particular window of ${\phi }_{SO}$. In particular, we choose ${\phi }_{SO}=0, \pi /4$ and $\pi/2$ . One can clearly see that for both ${\phi }_{SO}=\pi /4$ and $\pi /2$, ${G}_{\uparrow }$ and ${G}_{\downarrow }$ have an inverted behaviour as a function of $U$. For ${\phi }_{SO}=0,$ we find ${G}_{\uparrow }={G}_{\downarrow }$ which is, of course, an expected result. At around ${U}_{c}=2.6$, ${G}_{\sigma }$ has a discontinuity with respect to $U$ and with respect to ${\phi }_{SO},$ its sign reverses. To understand the discontinuity, we consider the second derivative of ${J}_{\sigma }$ with respect to ${\phi }_{SO}$.

9 $$\frac{{\partial^{2} J_{ \uparrow } }}{{\partial \phi_{SO}^{2} }} = {\mathcal{F}}_{1} \left( {\phi_{SO} } \right){\mathcal{G}}_{1} \left( {\phi_{SO} ,U} \right) + \left\{ {{\mathcal{F}}_{2} \left( {\phi_{SO} } \right){\mathcal{G}}_{2} \left( {\phi_{SO} ,U} \right) + {\mathcal{F}}_{3} \left( {\phi_{SO} } \right){\mathcal{G}}_{3} \left( {\phi_{SO} ,U} \right)} \right\},$$

10 $$\frac{{\partial^{2} J_{ \downarrow } }}{{\partial \phi_{SO}^{2} }} = {\mathcal{F}}_{1} \left( {\phi_{SO} } \right){\mathcal{G}}_{1} \left( {\phi_{SO} ,U} \right) - \left\{ {{\mathcal{F}}_{2} \left( {\phi_{SO} } \right){\mathcal{G}}_{2} \left( {\phi_{SO} ,U} \right) + {\mathcal{F}}_{3} \left( {\phi_{SO} } \right){\mathcal{G}}_{3} \left( {\phi_{SO} ,U} \right)} \right\},$$

where ${\mathcal{F}}^{\prime }$s are periodic functions of $\phi_{SO}$ and ${\mathcal{G}}^{\prime }$s are functions of Green’s functions. It may be noted that the Green functions appearing in the above equations change sign at a critical value of $U ({U}_{c})$ causing an overall change in both ${\partial }^{2}{J}_{\uparrow }/\partial {\phi }_{SO}^{2}$ and ${\partial }^{2}{J}_{\downarrow }/\partial {\phi }_{SO}^{2}$ at $U={U}_{c}$. Also, at $U={U}_{c},$ the positions of maxima and minima of ${J}_{\uparrow }$ and ${J}_{\downarrow }$ (with respect to ${\phi }_{SO})$ interchange. Hence, the gap between ${J}_{\uparrow }$ and ${J}_{\downarrow }$ at $U={U}_{c}$, becomes maximum. As ${G}_{\sigma }$ is directly related to ${J}_{\sigma }$, the interchange of maxima and minima of ${J}_{\uparrow }$ and ${J}_{\downarrow }$ causes a discontinuity at ${U}_{c}$ in the ${G}_{\sigma }$-spectrum.

### Spin-polarization

We study in this section the behaviour of the spin-polarization ${P}_{\uparrow ,\downarrow }$ (see Eq. 67) of a dissipative QDT system at finite temperature as a function of ${\phi }_{SO}$ in the presence of a magnetic field, e–p interaction, Coulomb correlation and quantum dissipation. ${P}_{\uparrow ,\downarrow }$ gives a measure of the spin-filtering effect that originates owing to the RSOI.

**Figure 15:**
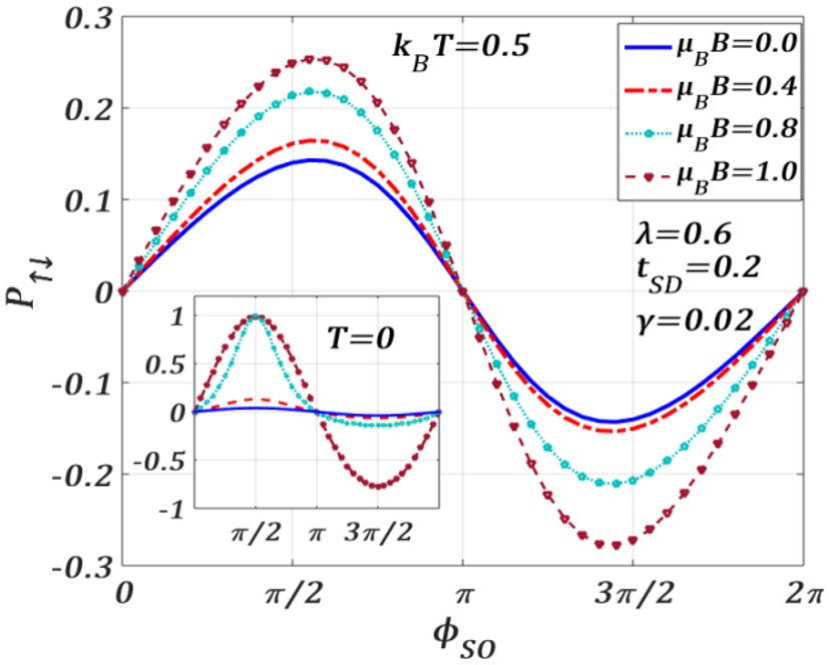
Spin-polarization ${P}_{\uparrow ,\downarrow }$ versus ${\phi }_{SO}$ for different ${\mu }_{B}B$ values for $\lambda =0.6,{t}_{SD}=0.2,\gamma =0.02, {eV}_{b}=0.5$ at ${k}_{B}T=0.5.$ Inset: at $T=0$.

Figure 15 describes the behaviour of ${P}_{\uparrow ,\downarrow }$ as a function of ${\phi }_{SO}$ for different values of $B$ with $\lambda = 0.6,{t}_{SD}=0.2,\gamma =0.02, {eV}_{b}=0.5,$ ${k}_{B}T=0.5$. ${P}_{\uparrow ,\downarrow }$ is positive in the region, $0\le {\phi }_{SO}\le \pi$ and negative in the region, $\pi \le {\phi }_{SO}\le 2\pi$ and is zero at ${\phi }_{SO}=0$, $\pi$ and $2\pi .$ Furthermore, $|{P}_{\uparrow ,\downarrow }|$ increases as magnetic field increases. Thus, the magnetic field favours spin-polarization. Also, the spin-polarization can be tuned by varying the strength of RSOI. Interestingly, the behaviour of ${P}_{\uparrow ,\downarrow }$ is not perfectly 2$\pi$-periodic at $B\ne 0$ as the magnetic field affects spin-up and spin-down electrons differently. At $T=0$ (see the inset), in the absence of the magnetic field, ${P}_{\uparrow ,\downarrow }$ remains essentially constant with ${\phi }_{SO}$. As $B$ increases, however, ${P}_{\uparrow ,\downarrow }$ does show a significant variation with ${\phi }_{SO}$ and the plots become asymmetric especially at ${\mu }_{B}B=0.8$ which is qualitatively different from that at a finite $T$. We find that the plots for ${\mu }_{B}B=0.5, 0.6, 0.7, 0.9$ also show a similar asymmetric behaviour. This issue can be explained as follows. At $T=0$, as the spin-polarization depends only on $B$ and ${\phi }_{SO}$ through the Green functions (Eq. 39), it is affected differently in different ranges of ${\phi }_{SO}$ for $B\ne 0.$ Because of the competition between B and ${\phi }_{SO},$ the plots show an asymmetric behaviour in the intermediate range of B and become more symmetric as B reaches a larger value. At finite T (main panel), $\mathrm{cos}({\phi }_{SO})$ term in Eq. (39) makes an additional contribution that makes the plots more symmetric. ${P}_{\uparrow ,\downarrow }$ exhibits a maximum at $\phi_{SO} = \pi /2$ ($P_{ \uparrow , \downarrow , max} = 1\;at\;\mu_{B} \;B = 1$) and a minimum at $\phi_{SO} = 3\pi /2$ ($P_{ \uparrow , \downarrow , min} = - 1\;{\text{approximately}}\;{\text{at}}\;\mu_{B} \;B = 1$). Therefore, it is possible to achieve a fully-polarized spin transport at $T=0$ with the help of a sufficiently high field. Once the maximum polarization is achieved at a particular ${\phi }_{SO}$, one can experimentally determine ${\alpha }_{R}$ for a given set of QDT parameters.

**Figure 16:**
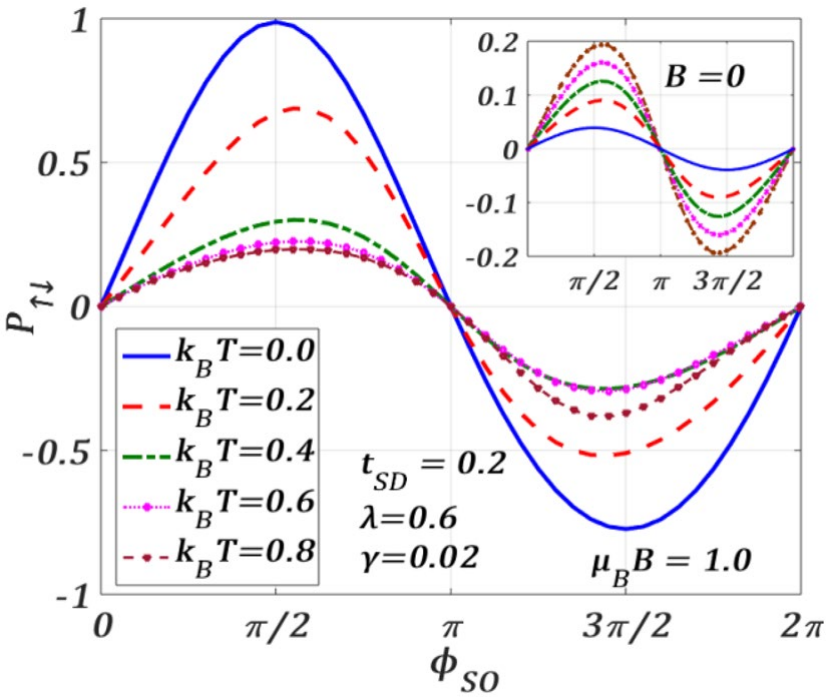
Spin-polarization ${P}_{\uparrow ,\downarrow }$ versus ${\phi }_{SO}$ for different ${k}_{B}T$ values for $\lambda =0.6,{t}_{SD}=0.2,\gamma =0.02, {eV}_{b}=0.5$ at $\mu_{B} \,B = 1.0.$ Inset: at $B=0$.

In Fig. 16, ${P}_{\uparrow ,\downarrow }$ is varied with ${\phi }_{SO}$ at a finite magnetic field in the regions $0\le {\phi }_{SO}\le \pi$ and $\pi \le {\phi }_{SO}\le 2\pi$ for different temperature values. In the region, $0\le {\phi }_{SO}\le \pi$, the polarization decreases with increasing temperature, while in the region $\pi \le {\phi }_{SO}\le 2\pi$, the magnitude of ${P}_{\uparrow ,\downarrow }$ decreases with increasing $T$ except for ${k}_{B}T=0.8$. Hence, a non-zero magnetic field can make the ${P}_{\uparrow ,\downarrow }$ variations non-monotonic with respect to $T$ for different ${\phi }_{SO}$. The inset show the plots for $B=0$. It is clear that, with respect to ${\phi }_{SO}$, ${P}_{\uparrow ,\downarrow }$ has a 2$\pi$-periodic variation for different values of $T$ and the behaviour is perfectly antisymmetric around ${\phi }_{SO}=\pi .$ Interestingly, in contrast to $B\ne 0,$ at $B=0$, temperature enhances $|{P}_{\uparrow ,\downarrow }|$, though the values of $|{P}_{\uparrow ,\downarrow }|$ are less than those at $B$. One may notice from the inset that ${P}_{\uparrow ,\downarrow , }=1$ cannot be achieved even at $T=0$ in the absence of the magnetic field. So, both the conditions of: $B\ne 0$ and $T=0$ are required to achieve complete polarization.

**Figure 17:**
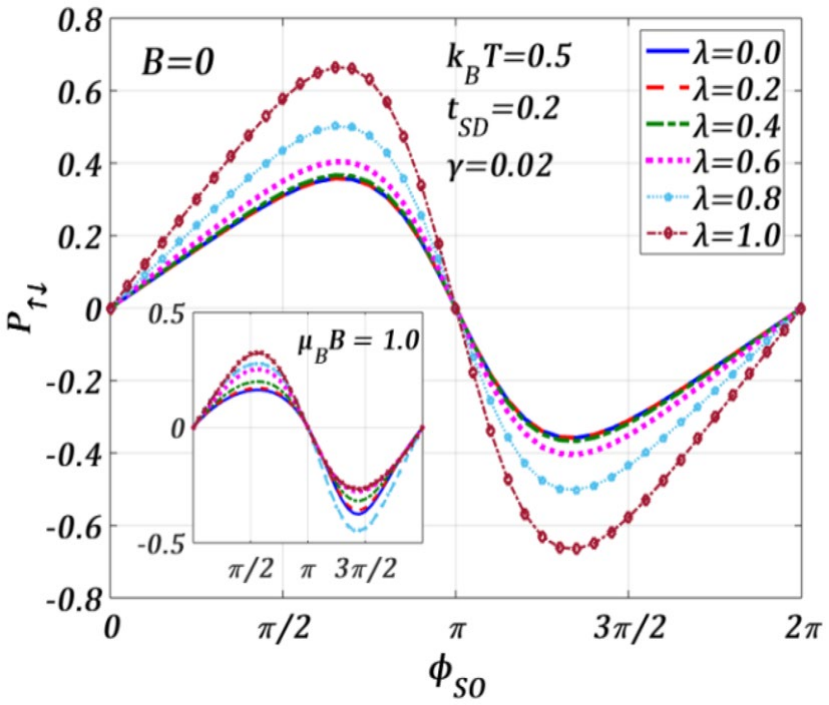
Spin-polarization ${P}_{\uparrow ,\downarrow }$ versus ${\phi }_{SO}$ for different $\lambda$ values at ${k}_{B}T=0.5$ ${t}_{SD}=0.2,\gamma =0.02, {eV}_{b}=0.5$ for $B=0$. Inset: at ${\mu }_{B}B=1.0$.

**Figure 18:**
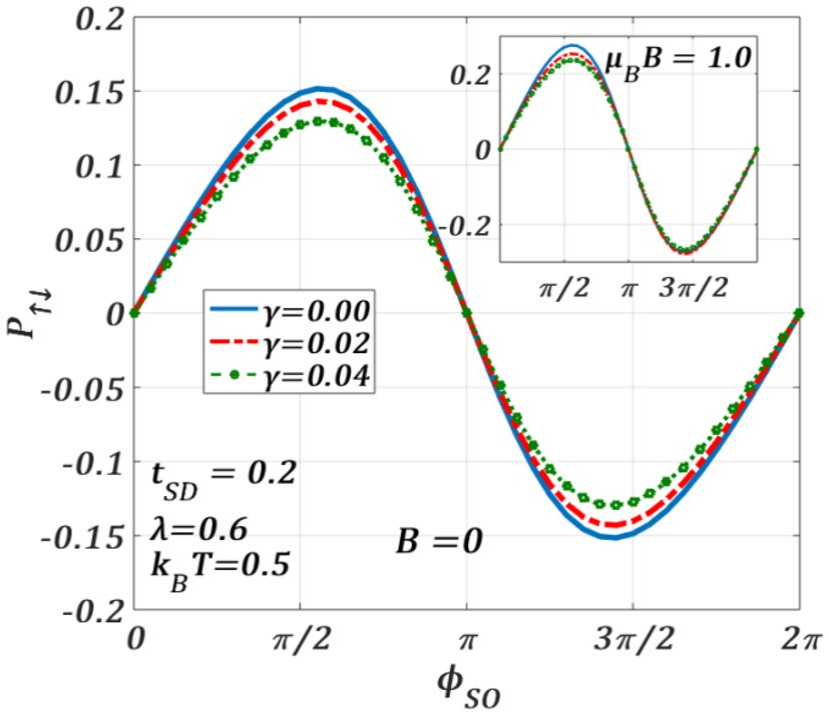
Spin-polarization ${P}_{\uparrow ,\downarrow }$ versus ${\phi }_{SO}$ for different $\gamma$ values at ${k}_{B}T=0.5$ ${t}_{SD}=0.2,\lambda =0.6, {eV}_{b}=0.5$ for $B=0$. Inset: at $\mu_{B} \,B = 1.0$.

In Fig. 17, we study the effect of e–p interaction on ${P}_{\uparrow ,\downarrow }$ both in the absence and presence of a magnetic field. It is observed that ${P}_{\uparrow ,\downarrow }$ shows a periodic pattern with a period $2\pi$. It is important to point out that the polaronic interaction increases the spin-polarization. The inset shows the behaviour at $B\ne 0$*.* As mentioned earlier, the magnetic field influences the spin-up and spin-down oppositely and therefore, the contrast in the variations of ${P}_{\uparrow ,\downarrow }$ is understandable. For completeness, we show the effect of dissipation on ${P}_{\uparrow ,\downarrow }$ in Fig. 18. Although $\gamma$ increases the tunneling spin currents ${J}_{\uparrow }$ and ${J}_{\downarrow }$, ${P}_{\uparrow ,\downarrow }$ reduces with increasing $\upgamma$ in both the regions: $0\le {\phi }_{SO}\le \pi$ and $\pi \le {\phi }_{SO}\le 2\pi$*.* The presence of a magnetic field (inset) makes the variations different both qualitatively and quantitatively. $|{P}_{\uparrow ,\downarrow ,max}|$ becomes larger in both the regions: $0\le {\phi }_{SO}\le \pi$ and $\pi \le {\phi }_{SO}\le 2\pi$***.*** Though the nature of the variations in the region: $0\le {\phi }_{SO}\le \pi$ remains essentially the same, in the region: $\pi \le {\phi }_{SO}\le 2\pi$*,* ${P}_{\uparrow ,\downarrow }$ seems to be independent of $\gamma .$

## Discussion

We have studied the effects of RSOI (measured by ${\phi }_{SO})$ on the non-equilibrium transport of a dissipative   QDT system where a single-level QD is embedded in a two-arm loop connected to two metallic leads so that transport occurs through two paths, one of which contains the QD. We consider the QD electrons to have the Holstein-Hubbard interactions and also the Rashba coupling. To reduce the effect of e–p coupling we introduce a dissipation term which can arise from the interaction of the QD phonon with the substrate phonons. This coupling is modelled by the linear Caldeira-Leggett Hamiltonian and the whole system is modelled by the Anderson-Holstein-Caldeira-Leggett Hamiltonian together with the RSOI and transport properties are calculated at finite temperature by Keldysh method. It is shown that without any external field, tunneling current gets decoupled completely by RSOI into spin-up and spin-down currents that are opposite in phase with respect to RSOI strengths. They are also $2\pi$-periodic with respect to ${\phi }_{SO}$ both in the absence and presence of the magnetic field. This SO interaction induced splitting between spin-up and spin-down currents and conductances can be tuned through the external gate voltage and magnetic field. We observe that the magnetic field influences the effects of e–p and RSO interactions on the spin-up and spin-down components differently. It also wipes out the phase correlation between the spin-up and spin-down conductances leading to complete separation of spin-up and spin-down spectra with no crossover. We also show that the dissipation originating from the QD-bath phonons interaction enhances the spin-resolved current, but the spin-polarization with respect to RSOI decreases with increasing dissipation in the absence of an external magnetic field. However, the change in the variations of spin-polarization is not significant as we turn on the magnetic field for the given set of parameters.

Though the e–p interaction usually restricts the flow of conduction electron owing to polaron formation, in the presence of RSOI, the spin-polarized conductances (${G}_{\uparrow }$ and ${G}_{\downarrow }$) do not always decrease with increasing $\lambda$ in the absence of the magnetic field. ${G}_{\uparrow }$ (${G}_{\downarrow }$) decreases (increases) with increasing $\lambda$ in the window: $0\le {\phi }_{SO}\le \pi$ and increases (decreases) with increasing $\lambda$ in the window: $\pi \le {\phi }_{SO}\le 2\pi$. There exists a phase correlation between ${G}_{\uparrow }$ and ${G}_{\downarrow }$ at zero magnetic field. Interestingly, in the presence of a magnetic field, this phase correlation is broken and ${G}_{\uparrow }$ reduces as $\lambda$ increases for all values of RSOI, but ${G}_{\downarrow }$ does not change much which again confirms that magnetic field acts differently on spin-up and spin-down components. This suggests that the effects of RSOI and e–p interaction on spin-transport get correlated through the external magnetic field. The spin resolved conductance is also $2\uppi$-periodic with respect to ${\phi }_{SO}$.

Finally, we have studied the variation of spin-polarization ${P}_{\uparrow ,\downarrow }$ as a function of RSOI for different ranges of the magnetic field, temperature, and e–p interaction. Like currents and conductances, the spin-polarization is also $2\pi$-periodic with respect to ${\phi }_{SO}$. We have shown that ${|P}_{\uparrow ,\downarrow }|$ increases with the external magnetic field at a finite temperature while it reduces with increasing temperature at a finite field. The polaronic interaction enhances the phenomenon of separation of up and down spins and consequently ${|P}_{\uparrow ,\downarrow }|$ increases significantly in the presence of e–p interaction. Our study predicts that though RSOI alone can produce a spin-filtering effect (without any external field), a fully spin-polarized (i.e., ${P}_{\uparrow ,\downarrow , max}=1$) transport can be achieved only at $T=0$ and a reasonably large magnetic field for a particular strength of RSOI. From the above conditions, one can determine experimentally the value of RSOI strength at which the maximum spin-polarization can occur.

Our results may find important applications in the fabrication of stronger spin-filtering devices in which the spin-filtering can be tuned by controlling the external magnetic field, RSOI and the e–p interaction in different temperature regimes.

## Methods

Before we present the detailed analytical technique of the Keldysh NEGF formalism for the calculation of the tunnelling current, we perform a series of transformations to decouple the interactions present in the system. To decouple SOI, we apply a transformation^44^ to $H$ by a unitary operator ${U}_{R}$ so that $H$ transforms to $\overline{H} = U_{R}^{\dag } H U_{R} .$ $U_{R}$ is chosen as

11 $$U_{R} = \left\{ \begin{gathered} 1\quad \quad \quad \quad \quad \quad \quad \quad {\text{for }}x < x_{S} , \hfill \\ \frac{1}{\sqrt 2 }e^{{ - ik_{R} \left( {x - x_{S} } \right)\sigma_{z} }} \quad \;\;{\text{for }}x_{S} < x < x_{D} , \hfill \\ \frac{1}{\sqrt 2 }e^{{ - ik_{R} \left( {x_{D} - x_{S} } \right)\sigma_{z} }} \quad {\text{for }}x_{D} < x. \hfill \\ \end{gathered} \right.$$

where $k_{R} = \left( {\alpha_{R} m^{*} /\hbar^{2} } \right)$. Defining a new set of operators*:* $\overline{c} = U_{R}^{\dag } c$ and $\overline{c} ^{\dag } = c^{\dag } U_{R}$, we can express $\overline{H}$ as

12 $$\begin{gathered} \overline{H} = \mathop \sum \limits_{k\sigma \in S,D} \varepsilon_{k} \left( {\overline{c}_{kS,\sigma }^{\dag } \overline{c}_{kS,\sigma } + \overline{c}_{kD,\sigma }^{\dag } \overline{c}_{kD,\sigma } } \right) + t_{SD } \mathop \sum \limits_{k\sigma \in S,D} \left( {\overline{c}_{kS,\sigma }^{\dag } \overline{c}_{kD,\sigma } + h.c.} \right) \hfill \\ \quad \quad \; + \mathop \sum \limits_{d\sigma } \overline{\varepsilon }_{d} \overline{n}_{d\sigma } + \mathop \sum \limits_{d} U\overline{n}_{d \uparrow } \overline{n}_{d \downarrow } + \left( {\frac{{p_{0}^{2} }}{{2m_{0} }} + \frac{1}{2}m_{0} \omega_{0}^{2} x_{0}^{2} } \right) + g\mathop \sum \limits_{d\sigma } \overline{n}_{d\sigma } x_{0} \hfill \\ \quad \quad \;\; + \frac{{\alpha_{R} }}{\hbar }\mathop \sum \limits_{{dd^{^{\prime}} }} \left[ {t_{{d^{\prime}d}}^{x} \left( {\overline{c}_{{d^{\prime}\sigma }}^{\dag } \overline{c}_{d\sigma } - \overline{c}_{{d^{\prime}, - \sigma }}^{\dag } \overline{c}_{d, - \sigma } } \right) + t_{{d^{\prime}d}}^{z} \left( {\overline{c}_{{d^{\prime}, - \sigma }}^{\dag } \overline{c}_{d\sigma } - \overline{c}_{d, - \sigma }^{\dag } \overline{c}_{{d^{\prime}\sigma }} } \right)} \right] + h.c. \hfill \\ \quad \quad \;\, + \mathop \sum \limits_{i = 1}^{N} \left[ {\frac{{p_{i}^{2} }}{{2m_{i} }} + \frac{1}{2}m_{i} \omega_{i}^{2} x_{i}^{2 } } \right] + \mathop \sum \limits_{i = 1}^{N} \beta_{i} x_{i} x_{0} \hfill \\ \quad \quad \; + \mathop \sum \limits_{kd\sigma } \left[ {V_{k} \left( {\overline{c}_{kS,\sigma }^{\dag } \overline{c}_{d\sigma } e^{{ - i\sigma k_{R} \left( {x - x_{S} } \right)}} + \overline{c}_{kD,\sigma }^{\dag } \overline{c}_{d\sigma } e^{{ - i\sigma k_{R} \left( {x - x_{D} } \right)}} } \right) + h.c} \right], \hfill \\ \end{gathered}$$

where ${\overline{\varepsilon }}_{d}=\left({\varepsilon }_{d}-e{V}_{\mathrm{g}}-\frac{1}{2}{g}^{*}{\mu }_{B}B{\sigma }_{z}\right)$. For simplicity, we assume that the QD contains effectively a single localized level and a single lattice mode which allows us to neglect the terms involving inter-level hopping and spin-flip term in the transformed Hamiltonian (12). Also, we choose $x=0$ and redefine: ${e}^{i{\sigma k}_{R}{x}_{S}}{\overline{c} }_{d\sigma } \mathrm{as} {c}_{d\sigma }$*.* The Hamiltonian $\overline{H }$ then reads

13 $$\begin{gathered} H = H_{S,D} + \mathop \sum \limits_{\sigma } \overline{\varepsilon }_{d} n_{d\sigma } + Un_{d\sigma } n_{d, - \sigma } + \left( {\frac{{p_{0}^{2} }}{{2m_{0} }} + \frac{1}{2}m_{0} \omega_{0}^{2} x_{0}^{2} } \right) + g\mathop \sum \limits_{\sigma } n_{d\sigma } x_{0} \hfill \\ \quad \quad \; + \mathop \sum \limits_{i = 1}^{N} \left[ {\frac{{p_{i}^{2} }}{{2m_{i} }} + \frac{1}{2}m_{i} \omega_{i}^{2} x_{i}^{2 } } \right] + \mathop \sum \limits_{i = 1}^{N} \beta_{i} x_{i} x_{0} \hfill \\ \quad \quad \; + \mathop \sum \limits_{k\sigma } \left[ {\left( {V_{k} c_{kS,\sigma }^{\dag } c_{d\sigma } + h.c} \right) + \left( {V_{k} c_{kD,\sigma }^{\dag } c_{d\sigma } e^{{ - i\sigma \phi_{SO} }} + h.c} \right)} \right], \hfill \\ \end{gathered}$$

which shows that the RSOI generates a spin-induced phase factor $-\sigma {\phi }_{SO}$ in the tunneling Hamiltonian for the (QD − D)—sector, where the SO phase ${\phi }_{SO}$ has been defined in Eq. (8).

Next, we proceed to deal with the interaction of the QD phonon with the bath phonons. To eliminate the substrate phonons partially, we perform the following canonical transformation^32,33,47^:

14 $${\widetilde{x}}_{i}=\left[{x}_{i}+\left(\frac{{\beta }_{i}}{{m}_{i}{\omega }_{i}^{2}}\right){ x}_{0}\right]; {\widetilde{p}}_{i}=-i \hslash \left(\frac{\partial }{\partial {\widetilde{x}}_{i}}\right),$$

which incorporates the most important aspect of the effect of the bath phonons which is dissipation. ${H}_{QD-B}$ reduces the QD phonon frequency which bringing in a frictional effect in the lattice mode of the QD. This is precisely the dissipative effect of the CL interaction and has been taken care of by the transformation (14). The frequency of the QD-phonon is modified as ${\widetilde{\omega }}_{0}={\left({\omega }_{0}^{2}-\Delta {\omega }^{2}\right)}^{1/2}$ , where $\Delta {\omega }^{2}$ is expressed as

15 $$\Delta {\omega }^{2}=\sum_{i=1}^{N}\frac{{\beta }_{i}^{2}}{{m}_{0}{m}_{i }{{\omega }_{i}}^{2}}$$

In the large $N$ limit, $\Delta {\omega }^{2}$ can be cast in an integral form through the spectral density function of the bath-phonon $I(\omega )$ over $\omega$ as

16 $$\Delta {\omega }^{2}=2\underset{0}{\overset{\infty }{\int }}\frac{I\left(\omega \right)}{{m}_{0}\omega } d\omega ,$$

where

17 $$I\left(\omega \right)=\sum_{i=1}^{N}\left[\frac{{\beta }_{i}^{2}}{2{m}_{i}{\omega }_{i}}\right]\delta \left(\omega -{\omega }_{i}\right),$$

which can be written in the Lorentz-Drude model as

18 $$I\left(\omega \right)= \frac{2{m}_{0}\gamma \omega }{\left[1+{\left(\frac{\omega }{{\omega }_{c}}\right)}^{2}\right]},$$

where $\gamma$ is the rate of quantum dissipation and ${\omega }_{c}$ is the cut-off frequency. As ${\omega }_{c}$ is considerably larger than other QDT frequencies, the deviation in the QD phonon frequency can be written as

19 $$\Delta {\omega }^{2}=2\pi \gamma {\omega }_{c}.$$

The interaction between the QD phonon and substrate phonons is partially decoupled and the higher-order terms are neglected for mathematical simplicity. The relevant QDT Hamiltonian reads

20 $$\begin{gathered} H = H_{S,D} + \mathop \sum \limits_{\sigma } \overline{\varepsilon }_{d} n_{d\sigma } + Un_{d,\sigma } n_{d, - \sigma } + \hbar \tilde{\omega }_{0} b^{\dag } b + \lambda \hbar \tilde{\omega }_{0} \left( {b^{\dag } + b} \right)\mathop \sum \limits_{\sigma } n_{d\sigma } \hfill \\ \quad \quad \; + \mathop \sum \limits_{k\sigma } \left[ {\left( {V_{k} c_{kS,\sigma }^{\dag } c_{d\sigma } + h.c} \right) + \left( {V_{k} c_{kD,\sigma }^{\dag } c_{d\sigma } e^{{ - i\sigma \phi_{SO} }} + h.c} \right)} \right], \hfill \\ \end{gathered}$$

where in the 5th term, $g$ together with all the multiplicative factors are clubbed into $\lambda$ as

21 $$\begin{gathered} g\mathop \sum \limits_{\sigma } n_{d\sigma } x_{0} = g\sqrt {\frac{\hbar }{{2m_{0} \omega_{0} }}} \left( {b^{\dag } + b} \right)\mathop \sum \limits_{\sigma } n_{d\sigma } = g\sqrt {\frac{1}{{2m_{0} \hbar \omega_{0} \tilde{\omega }_{0}^{2} }}} \hbar \tilde{\omega }_{0} \left( {b^{\dag } + b} \right)\mathop \sum \limits_{\sigma } n_{d\sigma } \hfill \\ \quad \quad \quad \quad \quad \; = \lambda \hbar \tilde{\omega }_{0} \left( {b^{\dag } + b} \right)\mathop \sum \limits_{\sigma } n_{d\sigma } , \hfill \\ \end{gathered}$$

where $\lambda =g{\left(1/2{m}_{0}\hslash {\omega }_{0}{{\widetilde{\omega }}_{0}}^{2}\right)}^{1/2}$ which we can refer to as the renormalized e–p interaction coefficient.

The next interaction to be dealt with is the e–p interaction. The e–p coupling can be removed by the well-known Lang-Firsov^51^ transformation:

22 $$e^{S} = \exp \left\{ {\lambda \mathop \sum \limits_{\sigma } n_{d\sigma } \left( {b^{\dag } - b} \right)} \right\}.$$

The transformed Hamiltonian can be expressed as

23 $$\begin{gathered} \tilde{H} = H_{S,D} + \mathop \sum \limits_{\sigma } \tilde{\varepsilon }_{d\sigma } n_{d\sigma } + \tilde{U}n_{d,\sigma } n_{d, - \sigma } + \hbar \tilde{\omega }_{0} b^{\dag } b \hfill \\ \quad \quad \; + \mathop \sum \limits_{k\sigma } \left[ {(\tilde{V}_{k} c_{kS,\sigma }^{\dag } c_{d\sigma } + h.c) + \left( {\tilde{V}_{k} c_{kD,\sigma }^{\dag } c_{d\sigma } e^{{ - i\sigma \phi_{SO} }} + h.c} \right)} \right], \hfill \\ \end{gathered}$$

where the phonon-mediated renormalized energy, modified Hubbard strength and the effective QD-lead hybridization strength are respectively given by

24 $${\widetilde{\varepsilon }}_{d\sigma }={\varepsilon }_{d}-e{\mathrm{\it V}}_{\mathrm{g}}-\sigma {\mu }_{B}B-{\lambda }^{2}\hslash {\widetilde{\omega }}_{0},$$

25 $$\widetilde{U}=U-2{\lambda }^{2}\hslash {\widetilde{\omega }}_{0},$$

26 $$\tilde{V}_{k} = e^{{ - \lambda \left( {b^{\dag } - b} \right)}} V_{k} = \widehat{\chi }V_{k} ; \widehat{\chi } = e^{{ - \lambda \left( {b^{\dag } - b} \right)}}$$

### Rashba induced spin-resolved tunneling via Keldysh method

Following Refs.^28,29^, the tunneling current from S to D through the QD embedded in the ring can be written as

27 $$J_{S\left( D \right)} = - e\langle\frac{{dN_{S\left( D \right)} }}{dt}\rangle = - \frac{ie}{\hbar }\langle\left[ {\tilde{H},\mathop \sum \limits_{k\sigma } c_{kS\left( D \right),\sigma }^{\dag } c_{kS\left( D \right),\sigma } } \right]\rangle,$$

where ${c}_{kS\left(D\right),\sigma }\left(t\right)={e}^{-iHt}{c}_{kS\left(D\right),\sigma }{e}^{iHt}$ and the averaging is to be done with respect to the actual ground state of the system $|0\rangle$ which is defined as $|0\rangle ={|0\rangle }_{el}{|0\rangle }_{ph}$. In the steady state, $J={J}_{S}=-{J}_{D}$ and after symmetrizing, we can write the tunneling current as

28 $${J}_{\sigma }=\frac{{J}_{S}-{J}_{D}}{2}\equiv \frac{e}{\hslash } Re\left\{\sum_{k}\langle {\widetilde{V}}_{k}\rangle {G}_{d\sigma ,kS}^{<} \left(t,t\right)-\sum_{k}\langle {{\widetilde{V}}_{k}}^{\sigma }\rangle {G}_{d\sigma ,kD}^{<} \left(t,t\right)\right\},$$

where ${\widetilde{V}}_{k}$ has been defined earlier, ${{\widetilde{V}}_{k}}^{\sigma }={\widetilde{V}}_{k}{e}^{-i{\sigma \phi }_{SO}},$ $\langle \dots \rangle$ denotes the expectation value of … with respect to $n$ th-phonon state i.e., $\langle {\widetilde{V}}_{k}\rangle =\langle n|{\widetilde{V}}_{k}|n\rangle$ and $\langle {{\widetilde{V}}_{k}}^{\sigma }\rangle =\langle n|{{\widetilde{V}}_{k}}^{\sigma }|n\rangle$ and ${G}_{d\sigma ,kS\left(D\right)}^{<}\left(t,{t}^{^{\prime}}\right)$ and ${G}_{d\sigma ,kS\left(D\right)}^{>}\left(t,{t}^{^{\prime}}\right)$ are respectively the lesser and the greater (tunneling) Keldysh Green functions defined as

29 $$G_{d\sigma ,kS\left( D \right)}^{ < } \left( {t,t^{\prime}} \right) = i\langle0\left| {c_{kS\left( D \right)}^{\dag } \left( {t^{\prime}} \right)c_{d\sigma } \left( t \right)} \right|0\rangle,$$

30 $$G_{d\sigma ,kS\left( D \right)}^{ > } \left( {t,t^{\prime}} \right) = - i\langle0\left| {c_{d\sigma } \left( {t^{\prime}} \right)c_{kS\left( D \right)}^{\dag } \left( t \right)} \right|0\rangle.$$

Now, we define the retarded ($r$) and advanced ($a$) tunneling Green functions ${G}_{d\sigma ,kS(D)}^{r(a)} \left(t,{t}^{^{\prime}}\right)$ as

31 $$G_{d\sigma ,kS\left( D \right)}^{r\left( a \right)} { }\left( {t,t^{\prime}} \right) = \mp i\theta \left( { \pm t \mp t^{\prime}} \right)\langle0\left| {\left\{ {\tilde{c}_{d\sigma } \left( t \right),c_{kS,\sigma }^{\dag } \left( {t^{\prime}} \right)} \right\}} \right|0\rangle,$$

where ${c}_{d\sigma }\left(t\right)={e}^{-i{\tilde{H}}_{el}t}{c}_{d\sigma }{e}^{i{\tilde{H}}_{el}t}$ and ${\widetilde{c}}_{d\sigma }\left(t\right)=\widehat{\chi }{c}_{d\sigma }(t).$ Using the equation of motion of ${G}_{d\sigma ,kS(D)}^{r(a)}\left(t,{t}^{^{\prime}}\right)$ and applying the analytical continuation rule of Langreth, we get the expression for ${G}_{d\sigma ,kS(D)}^{<}\left(t,{t}^{^{\prime}}\right)$ as

32 $${G}_{d\sigma ,kS}^{<}\left(t,{t}^{^{\prime}}\right)=\int \frac{d\omega }{2\pi }\left[{{V}_{k}}^{*}+{{{V}_{k}}^{\sigma }}^{*}{t}_{SD}\right]\left[{G}_{dd}^{<}\left(\omega \right){g}_{kS}^{a}\left(\omega \right)+{G}_{dd}^{r}\left(\omega \right){g}_{kS}^{<}\left(\omega \right)\right]{e}^{-i\omega (t-{t}^{^{\prime}})},$$

33 $${G}_{d\sigma ,kD}^{<}\left(t,{t}^{^{\prime}}\right)=\int \frac{d\omega }{2\pi }\left[{{{V}_{k}}^{\sigma }}^{*}+{{V}_{k}}^{*}{t}_{SD}\right]\left[{G}_{dd}^{<}\left(\omega \right){g}_{kD}^{a}\left(\omega \right)+{G}_{dd}^{r}\left(\omega \right){g}_{kD}^{<}\left(\omega \right)\right]{e}^{-i\omega (t-{t}^{^{\prime}})},$$

where ${g}_{kS\left(D\right)}^{r\left(a\right)}\left(\omega \right)$ and ${g}_{kS(D)}^{<}\left(\omega \right)$ are the lead Green functions in the energy space which are related by Fourier transformation (FT) to the corresponding time-dependent Green functions ${g}_{kS\left(D\right)}^{r\left(a\right)} \left(t,{t}^{^{\prime}}\right)$ and ${g}_{kS\left(D\right)}^{<} \left(t,{t}^{^{\prime}}\right)$ defined by

34 $$g_{kS\left( D \right)}^{r\left( a \right)} { }\left( {t,t^{\prime}} \right) = \mp i\theta \left( { \pm t \mp t^{\prime}} \right)\langle0\left| {\left\{ {c_{kS\left( D \right),\sigma } \left( t \right),c_{kS\left( D \right),\sigma }^{\dag } \left( {t^{\prime}} \right)} \right\}} \right|0\rangle,$$

35 $$g_{kS\left( D \right)}^{ < } { }\left( {t,t^{\prime}} \right) = i{ }\langle c_{kS\left( D \right),\sigma }^{\dag } \left( {t^{\prime}} \right)c_{kS\left( D \right),\sigma } \left( t \right)\rangle,$$

${G}_{dd}^{r(a)}\left(\omega \right)$ and ${G}_{dd}^{<(>)}\left(\omega \right)$ are the energy-dependent retarded (advanced) and the Keldysh lesser(greater) Green functions of the QD which can be obtained by Fourier transforming the corresponding time-dependent Green functions ${G}_{dd}^{r\left(a\right)}\left(t,{t}^{^{\prime}}\right)$ and ${G}_{dd}^{<(>)}\left(\tau =t-{t}^{^{\prime}}\right)$ defined respectively by

36 $$G_{dd}^{r\left( a \right)} \left( {t,t^{\prime}} \right) = \mp i{ }\theta \left( { \pm t \mp t^{\prime}} \right)\langle0\left| {\left\{ {\tilde{c}_{d\sigma } \left( t \right),\tilde{c}_{d\sigma }^{\dag } \left( {t^{\prime}} \right)} \right\}} \right|0\rangle,$$

and

37 $$G_{dd}^{ < } \left( \tau \right) = i\langle0{|}\tilde{c}_{d\sigma }^{\dag } \left( 0 \right)\tilde{c}_{d\sigma } \left( \tau \right){|}0\rangle,$$

38 $$G_{dd}^{ > } \left( \tau \right) = - i\langle0{|}\tilde{c}_{d\sigma } \left( \tau \right)\tilde{c}_{d\sigma }^{\dag } \left( 0 \right){|}0\rangle.$$

Substituting Eqs. (32) and (33) together with (34) and (35) in Eq. (28), we get an expression of ${J}_{\sigma }$ which after some algebraic manipulations becomes

39 $$\begin{gathered} J_{\sigma } = \frac{e}{2h}\it\Gamma \left[ {\left( {1 + t_{SD} \cos \left( {\sigma \phi_{SO} } \right)} \right)\smallint \frac{d\omega }{{2\pi }} \left( {f_{S} \left( \omega \right) - f_{D} \left( \omega \right)} \right)A\left( \omega \right)} \right. \hfill \\ \quad \quad \; - t_{SD} \sin \left( {\sigma \phi_{SO} } \right)\smallint \frac{d\omega }{{2\pi }} \left( {f_{S} \left( \omega \right) + f_{D} \left( \omega \right)} \right) \left( {G_{dd}^{r} \left( \omega \right) + G_{dd}^{a} \left( \omega \right)} \right) \hfill \\ \quad \quad \left. {\; - 4t_{SD} \sin \left( {\sigma \phi_{SO} } \right)\smallint \frac{d\omega }{{2\pi }} Re\left\{ { G_{dd}^{ < } \left( \omega \right)} \right\}} \right], \hfill \\ \end{gathered}$$

where ${f}_{S,D}\left(\varepsilon \right)={\left(exp[({\mu }_{S,D}-\varepsilon )/{k}_{B}T]+1 \right)}^{-1}$ are the Fermi functions for S and D, ${\mu }_{S,D}$ being the corresponding chemical potentials which are related through ${V}_{b}$ and ${V}_{m}$ as: ${\mu }_{S}=e{V}_{m}+e{V}_{b}/2, {\mu }_{D}=e{V}_{m}-e{V}_{b}/2$, $\it\Gamma ={(\it\Gamma }_{S}+{\it\Gamma }_{D})/2$, where ${\it\Gamma }_{S}$ and ${\it\Gamma }_{D}$ are defined as

40 $${\it\Gamma }_{S,D}=\it\Gamma =2\pi {\rho }_{S,D}\langle {\widetilde{V}}_{k}\rangle {V}_{k}^{*},$$

where ${\rho }_{S,D}$ being the density of states of leads and $\langle {\widetilde{V}}_{k}\rangle$ can be expressed as

41 $$\langle\tilde{V}_{k}\rangle { } = \langle n\left| {\tilde{V}_{k} } \right|n\rangle = V_{k}\langle n\left| {e^{{ - \lambda \left( {b^{\dag } - b} \right)}} } \right|n\rangle = V_{k} \frac{{\langle n\left| {\mathop \sum \nolimits_{n = 0}^{\infty } e^{{ - n\hbar \tilde{\omega }_{0} /k_{B} T}} e^{{ - \lambda \left( {b^{\dag } - b} \right)}} } \right|n\rangle}}{{\langle n\left| {\mathop \sum \nolimits_{n = 0}^{\infty } e^{{ - n\hbar \tilde{\omega }_{0} /k_{B} T}} } \right|n\rangle}} = V_{k} e^{{ - \lambda^{2} \left( {f_{ph} + 1/2} \right)}} .$$

$A\left(\omega \right)$ is the spectral function (SF) of the QDT system which can be expressed as

42 $$A\left( \omega \right) = i\left[ {G_{dd}^{r} \left( \omega \right) - G_{dd}^{a} \left( \omega \right)} \right] = { }i\left[ {G_{dd}^{ > } \left( \omega \right) - G_{dd}^{ < } \left( \omega \right)} \right],$$

To calculate $A\left(\omega \right)$ and hence ${J}_{\sigma }$, we need to calculate ${G}_{dd}^{r\left(a\right)}(\omega )$ and ${G}_{dd}^{<(>)}\left(\omega \right)$. ${G}_{dd}^{r\left(a\right)}\left(t,{t}^{^{\prime}}\right)$ can be written as

43 $$G_{dd}^{r\left( a \right)} \left( {t,t^{\prime}} \right) = \left[ {\tilde{G}_{dd}^{r\left( a \right)} \left( {t,t^{\prime}} \right)} \right]_{el}\langle \widehat{{\chi { }}}\left( t \right)\hat{\chi }^{\dag } \left( {t^{\prime}} \right)\rangle_{ph} = \left[ {\tilde{G}_{dd}^{r\left( a \right)} \left( {t,t^{\prime}} \right)} \right]_{el} e^{ - \varphi \left( \tau \right)} ,$$

where $\left[ {\tilde{G}_{dd}^{r\left( a \right)} \left( {t,t^{\prime}} \right)} \right]_{el}$ is defined as

44 $$\left[ {\tilde{G}_{dd}^{r\left( a \right)} \left( {t,t^{\prime}} \right)} \right]_{el} = \mp i{ }\theta \left( { \pm t \mp t^{\prime}} \right)\langle0\left| {\left\{ {c_{d\sigma } \left( t \right),c_{d\sigma }^{\dag } \left( {t^{\prime}} \right)} \right\}} \right|0\rangle_{el} ,$$

and $\langle\widehat{\chi }\left( t \right)\hat{\chi }^{\dag } \left( {t^{\prime}} \right)\rangle_{ph}$ is calculated as

45 $$\langle\widehat{{\chi { }}}\left( t \right)\hat{\chi }^{\dag } \left( {t^{\prime}} \right)\rangle_{ph} { } = \langle e^{{ - i\tilde{H}_{ph} t}} \widehat{{\chi { }}}e^{{i\tilde{H}_{ph} t}} e^{{ - i\tilde{H}_{ph} t^{\prime}}} \hat{\chi }^{\dag } e^{{i\tilde{H}_{ph} t^{\prime}}}\rangle_{ph} = e^{ - \varphi \left( \tau \right)} ,$$

with

46 $$\varphi \left(\tau \right)={\lambda }^{2}\left[2{f}_{ph}+1-2{\left\{{f}_{ph}\left(1+{f}_{ph}\right)\right\}}^{1/2}cos\left(\hslash {\widetilde{\omega }}_{0}\left(\tau +i\beta /2\right)\right)\right],$$

where ${f}_{ph}$ is the phonon distribution function given by ${f}_{ph}={\left[exp\left(\hslash {\widetilde{\omega }}_{0}/{k}_{B}T\right)-1\right]}^{-1}$. After some algebraic manipulation, we obtain

47 $$\varphi \left(\tau \right)=- \mathrm{ln}\left[\sum_{n=-\infty }^{\infty }{L}_{n}\left(z\right){e}^{-in\hslash {\widetilde{\omega }}_{0}\tau }\right],$$

where ${L}_{n}$ is the spectral weight of the $n$th phonon side band^30^ and is given by

48 $${L}_{n}\left(z\right)=\mathit{exp}\left[-{\lambda }^{2}\left(2{f}_{ph}+1\right)+\left(\frac{n\hslash {\widetilde{\omega }}_{0}}{2{k}_{B}T}\right)\right]{I}_{n}\left(z\right),$$

where $z=2{\lambda }^{2}{\left[{f}_{ph}\left(1+{f}_{ph}\right)\right]}^\frac{1}{2}$, $n$ is the number of phonons and ${I}_{n}$ is the Modified Bessel function of second kind. Thus, ${G}_{dd}^{r(a)}\left(\omega \right)$ can be written in the $\omega$-space as

49 $${G}_{dd}^{r(a)}\left(\omega \right)=\sum_{n=-\infty }^{\infty }{L}_{n}(z){\left[{\widetilde{G}}_{dd}^{r\left(a\right)}\left(\omega -n\hslash {\widetilde{\omega }}_{0}\right)\right]}_{el} ,$$

where the Green functions ${\left[{\widetilde{G}}_{dd}^{r,a}\left(\omega \right)\right]}_{el}$ are the FTs of ${\left[{\widetilde{G}}_{dd}^{r\left(a\right)}\left(t,{t}^{^{\prime}}\right)\right]}_{el}$ in the $\omega -$ space.

Using the equation of motion method^28^ and employing the mean-field Hartree–Fock approximation for the onsite Coulomb term, ${\left[{\widetilde{G}}_{dd}^{r\left(a\right)}\right]}_{el}$ is obtained in $\omega$-space as

50 $${\left[{\widetilde{G}}_{dd}^{r(a)}\left(\omega \mp n\hslash {\widetilde{\omega }}_{0}\right)\right]}_{el}={\left[\omega \mp n\hslash {\widetilde{\omega }}_{0}-{\widetilde{\varepsilon }}_{d\sigma }-\widetilde{U}\langle {n}_{d,-\sigma }\rangle -{\widetilde{\sum }}^{r\left(a\right)}(\omega )\right]}^{-1},$$

where $\langle {n}_{d,-\sigma }\rangle$ is the average occupancy of the QD electron number and ${\widetilde{\sum }}^{r\left(a\right)}(\omega )$ is the retarded (advanced) self-energy which reads

51 $${\widetilde{\sum }}^{r\left(a\right)}\left(\omega \right)=\sum_{k}\left(\frac{{\left|<{\widetilde{V}}_{k}>\right|}^{2}\left(\omega \mp n\hslash {\widetilde{\omega }}_{0}-{\varepsilon }_{k}+{t}_{SD}\mathit{cos}{\phi }_{SO}\right)}{\left(\omega \mp n\hslash {\widetilde{\omega }}_{0}-{\varepsilon }_{k}+{t}_{SD}\right)\left(\omega \mp n\hslash {\widetilde{\omega }}_{0}-{\varepsilon }_{k}-{t}_{SD}\right)}\right) =\widetilde{\Lambda }\left(\omega \right)\mp i\widetilde{\Gamma }\left(\omega \right),$$

where the real part of ${\widetilde{\sum }}^{r\left(a\right)}(\omega )$ can be clubbed with the QD energy and the imaginary part assumes the following expression

52 $$\widetilde{\it\Gamma }= \it\Gamma {e}^{-{\lambda }^{2}\left({f}_{ph}+1/2\right)}.$$

Thus, using Eq. (42), $A\left(\omega \right)$ can be expressed as

53 $$A\left(\omega \right)=\sum_{n=-\infty }^{\infty }2\widetilde{\it\Gamma }{L}_{n}\left(z\right){\left[{\left(\omega \mp n\hslash {\widetilde{\omega }}_{0}-{\widetilde{\varepsilon }}_{d\sigma }-\widetilde{U}\langle {n}_{d,-\sigma }\rangle \right)}^{2}+{\widetilde{\it\Gamma }}^{2}\right]}^{-1}.$$

Following the same procedure as above, $\langle {n}_{d\sigma }\rangle$ can be obtained for a symmetric QDT, as

54 $$\langle {n}_{d\sigma }\rangle =\frac{1}{2\pi }\int d\omega \left[{f}_{s}\left(\omega \right)+{f}_{D}\left(\omega \right)\right]A\left(\omega \right).$$

$A\left(\omega \right)$ can be determined by self-consistently solving Eqs. (53) and (54). To obtain ${J}_{\sigma }$, we also need to calculate ${G}_{dd}^{<(>)}\left(\omega \right)$ which can be determined from ${G}_{dd}^{<}\left(\tau =t-{t}^{^{\prime}}\right)$ and ${G}_{dd}^{>}\left(\tau =t-{t}^{^{\prime}}\right)$ by FT. ${G}_{dd}^{<(>)}\left(\tau \right)$ can be expanded as

55 $$G_{dd}^{ < } \left( \tau \right) = i\langle0{|}c_{d}^{\dag } \left( 0 \right)c_{d} \left( \tau \right){|}0\rangle_{el}\langle \hat{\chi }^{\dag } \left( 0 \right)\widehat{{\chi { }}}\left( \tau \right)\rangle_{ph} = \tilde{G}_{dd}^{ < } \left( \tau \right)_{el} \mathop \sum \limits_{n = - \infty }^{\infty } L_{n} e^{{in\hbar \tilde{\omega }_{0} \tau }} ,$$

56 $$G_{dd}^{ > } \left( \tau \right) = - i\langle0{|}c_{d} \left( \tau \right)c_{d}^{\dag } \left( 0 \right){|}0\rangle_{el}\langle \widehat{{\chi { }}}\left( \tau \right)\hat{\chi }^{\dag } \left( 0 \right)\rangle_{ph} = \tilde{G}_{dd}^{ > } \left( \tau \right)_{el} \mathop \sum \limits_{n = - \infty }^{\infty } L_{n} e^{{ - in\hbar \tilde{\omega }_{0} \tau }} ,$$

where

57 $$\tilde{G}_{dd}^{ < } \left( \tau \right) = i\langle0{|}c_{d}^{\dag } \left( 0 \right)c_{d} \left( \tau \right){|}0\rangle_{el} = i\langle0{|}c_{d}^{\dag } \left( 0 \right)e^{{ - i\tilde{H}_{el} \tau }} c_{d} e^{{i\tilde{H}_{el} \tau }} { |}0\rangle_{el} ,$$

58 $$\tilde{G}_{dd}^{ > } \left( \tau \right) = - i\langle0{|}c_{d} \left( \tau \right)c_{d}^{\dag } \left( 0 \right){|}0\rangle_{el} = - i\langle0{|}e^{{ - i\tilde{H}_{el} \tau }} c_{d} e^{{i\tilde{H}_{el} \tau }} c_{d}^{\dag } \left( 0 \right){ |}0\rangle_{el} ,$$

and

59 $$\langle\hat{\chi }^{\dag } \left( 0 \right)\widehat{{\chi { }}}\left( \tau \right)\rangle_{ph} = \langle\hat{\chi }^{\dag } \left( 0 \right)e^{{ - i\tilde{H}_{ph} \tau }} \widehat{{\chi { }}}e^{{i\tilde{H}_{ph} \tau }}\rangle_{ph} = \mathop \sum \limits_{n = - \infty }^{\infty } L_{n} e^{{in\hbar \tilde{\omega }_{0} \tau }} ,$$

60 $$\langle\widehat{{\chi { }}}\left( \tau \right)\hat{\chi }^{\dag } \left( 0 \right)\rangle_{ph} =\langle e^{{ - i\tilde{H}_{ph} \tau }} \widehat{{\chi { }}}e^{{i\tilde{H}_{ph} \tau }} \hat{\chi }^{\dag } \left( 0 \right)\rangle_{ph} = \mathop \sum \limits_{n = - \infty }^{\infty } L_{n} e^{{ - in\hbar \tilde{\omega }_{0} \tau }} ,$$

${G}_{dd}^{<}\left(\omega \right)$ and ${G}_{dd}^{>}\left(\omega \right)$ are now obtained as

61 $${G}_{dd}^{<}\left(\omega \right)=\sum_{n=-\infty }^{\infty }{L}_{n}{\widetilde{G}}_{dd}^{<}\left(\omega +n\hslash {\widetilde{\omega }}_{0}\right),$$

62 $${G}_{dd}^{>}\left(\omega \right)=\sum_{n=-\infty }^{\infty }{L}_{n}{\widetilde{G}}_{dd}^{>}\left(\omega -n\hslash {\widetilde{\omega }}_{0}\right).$$

where ${\widetilde{G}}_{dd}^{<}\left(\omega \right)$ and ${\widetilde{G}}_{dd}^{>}\left(\omega \right)$ are the FTs of ${\widetilde{G}}_{dd}^{<}\left(\tau \right)$ and ${\widetilde{G}}_{dd}^{>}\left(\tau \right)$ respectively, in the $\omega -$ space.

By applying the Langreth’s analytical continuation rule to the Dyson equations for ${\widetilde{G}}^{<\left(>\right)},$ we can show that ${\widetilde{G}}^{<\left(>\right)}$ satisfies the equation

63 $${\widetilde{G}}^{<\left(>\right)}\left(\omega \right)={\widetilde{G}}_{dd}^{r}\left(\omega \right){ \widetilde{\Sigma }}^{<\left(>\right)}\left(\omega \right) {\widetilde{G}}_{dd}^{a}\left(\omega \right),$$

where ${\widetilde{G}}_{dd}^{r(a)}\left(\omega \right)$ is given by Eq. (50) and the lesser and greater self-energies are obtained as

64 $${\widetilde{\Sigma }}^{<}\left(\omega \right)=i \widetilde{\Gamma }\left[{f}_{S}\left(\omega \right)+{f}_{D}\left(\omega \right)\right],$$

and

65 $${\widetilde{\Sigma }}^{>}\left(\omega \right)=-i \widetilde{\Gamma } \left[2-{(f}_{S}\left(\omega \right)+{f}_{D}\left(\omega \right))\right].$$

Substituting Eqs. (63) together with (64) and (65) in Eqs. (61) and (62) we can then obtain ${G}^{<\left(>\right)}\left(\omega \right).$ Once ${G}^{<\left(>\right)}\left(\omega \right),$ ${G}_{dd}^{r(a)}\left(\omega \right)$ and $A(\omega )$ are obtained, the tunneling current ${J}_{\sigma }$ can be computed using Eq. (39).

We would like to mention that the derivations of Eqs. (32) and (33) are made under the assumptions: ${\left[{g}_{kS\left(D\right)}^{r\left(a\right)} \left(t,{t}^{^{\prime}}\right)\right]}^{2}\approx {g}_{kS\left(D\right)}^{r\left(a\right)} \left(t,{t}^{^{\prime}}\right)$ and ${t}_{SD}\ll {V}_{k},$ so that the terms of order higher than ${t}_{SD}{g}_{kS\left(D\right)}^{r\left(a\right)}$ can be neglected. As we have already mentioned earlier, there exist two different paths for the metallic electrons to tunnel from S to D, one through a QD with SOI and the other directly by hopping from S to D. Thus, the SO phase ${\phi }_{SO}$ expressed in Eq. (8) is essentially the phase difference between two paths*.* We finally calculate the spin-resolved differential conductance ${G}_{\sigma }$ and the spin-polarization ${P}_{\sigma ,-\sigma }$ which are defined respectively as:

66 $${G}_{\sigma }=\frac{d{J}_{\sigma }}{{dV}_{b}},$$

67 $${P}_{\sigma ,-\sigma }=\frac{{J}_{\sigma }-{J}_{-\sigma }}{{J}_{\sigma }+{J}_{-\sigma }}.$$

## Data Availability

The datasets used and/or analysed during the current study available from the corresponding author on reasonable request.
